# CalTrack: High-Throughput Automated Calcium Transient Analysis in Cardiomyocytes

**DOI:** 10.1161/CIRCRESAHA.121.318868

**Published:** 2021-05-21

**Authors:** Yiangos Psaras, Francesca Margara, Marcelo Cicconet, Alexander J. Sparrow, Giuliana G. Repetti, Manuel Schmid, Violetta Steeples, Jonathan A.L. Wilcox, Alfonso Bueno-Orovio, Charles S. Redwood, Hugh C. Watkins, Paul Robinson, Blanca Rodriguez, Jonathan G. Seidman, Christine E. Seidman, Christopher N. Toepfer

**Affiliations:** 1 Division of Cardiovascular Medicine, Radcliffe Department of Medicine (Y.P., F.M., A.J.S., M.S., V.S., C.S.R., H.C.W., P.R., C.N.T.), University of Oxford, United Kingdom.; 2 Computer Science (F.M., A.B.-O., B.R.), University of Oxford, United Kingdom.; 3 Wellcome Centre for Human Genetics (H.C.W., C.N.T.), University of Oxford, United Kingdom.; 4 Image and Data Analysis Core (M.C.), Harvard Medical School, Boston, MA; 5 Genetics (G.G.R., J.A.L.W., J.G.S., C.E.S., C.N.T.), Harvard Medical School, Boston, MA; 6 Cardiovascular Division, Department of Medicine, Brigham and Women’s Hospital, Boston, MA (C.E.S.).; 7 Howard Hughes Medical Institute, Chevy Chase, MD (C.E.S.).

**Keywords:** calcium, cardiomyopathy, hypertrophic, induced pluripotent stem cells, myocytes, cardiac, photobleaching

## Abstract

Calcium transient analysis is central to understanding inherited and acquired cardiac physiology and disease. Although the development of novel calcium reporters enables assays of CRISPR/Cas-9 genome-edited induced pluripotent stem cell–derived cardiomyocytes and primary adult cardiomyocytes, existing calcium-detection technologies are often proprietary and require specialist equipment, whereas open-source workflows necessitate considerable user expertise and manual input.

**Objective:**

We aimed to develop an easy to use open-source, adaptable, and automated analysis pipeline for measuring cellular calcium transients, from image stack to data output, inclusive of cellular identification, background subtraction, photobleaching correction, calcium transient averaging, calcium transient fitting, data collation, and aberrant behavior recognition.

**Methods and Results:**

We developed CalTrack, a MatLab-based algorithm, to monitor fluorescent calcium transients in living cardiomyocytes, including isolated single cells or those contained in 3-dimensional tissues or organoids, and to analyze data acquired using photomultiplier tubes or employing line scans. CalTrack uses masks to segment cells allowing multiple cardiomyocyte transients to be measured from a single field of view. After automatically correcting for photobleaching, CalTrack averages and fits a string of transients and provides parameters that measure time to peak, time of decay, tau, peak fluorescence/baseline fluorescence (F_max_/F_0_), and others. We demonstrate the utility of CalTrack in primary and induced pluripotent stem cell–derived cell lines in response to pharmacological compounds and in phenotyping cells carrying hypertrophic cardiomyopathy variants.

**Conclusions:**

CalTrack, an open-source tool that runs on a local computer, provides automated high-throughput analysis of calcium transients in response to development, genetic or pharmacological manipulations, and pathological conditions. We expect that CalTrack analyses will accelerate insights into physiological and abnormal calcium homeostasis that influence diverse aspects of cardiomyocyte biology.

## Abstract 2

Supplemental Digital Content is available in the text.

**Meet the First Author, see p 220**

Calcium is a universal signaling molecule that can evoke extensive changes in the proteome and transcriptome of all cells and has particular roles in excitable neural cells and cardiomyocytes. In addition to cell signaling, calcium transients in cardiomyocytes can directly influence contraction, relaxation, and arrhythmogenicity. As such, dynamic calcium measurements are key to understanding cardiomyocyte physiology, pathology, and the therapeutic efficacy and safety in delivered compounds.^1^

Investigations into cardiovascular biology employ fluorescent indicators of cellular calcium, including cell-permeable fluorophores,^2,3^ and viral delivery of genetically encoded calcium sensors^4,5^ that may target subcellular structures such as sarcomeres.^6,7^ Despite the successful application of these strategies to primary cells derived from experimental models or explanted human tissues,^8^ there remains an unmet need for higher throughput calcium screening assays, in particular, to address human immortalized embryonic stem cell and induced pluripotent stem cell–derived cardiomyocytes (iPSC-CMs). With this goal, we developed CalTrack, a set of MatLab algorithms with the flexibility to analyze cellular calcium transients from many cellular formats in an unbiased, rapid, analysis platform. CalTrack uses high-throughput fluorescent cellular imaging to enable analyses of wild-type (WT)^9^ and mutant iPSC-CMs derived from patients or mutated using clustered regularly interspaced short palindromic repeats (CRISPR)/Cas-9 methodologies.^10^

Phenotyping of iPSC-CMs has recently become easier with techniques that allow rapid automated assessments of contractile function^11,12^ and that address challenges of cellular heterogeneity by increasing experimental throughput, as greater data volume enables statistical power to interrogate small effects. Although the development of more physiologically relevant models remains an important endeavor to overcome cellular immaturity, rapid phenotyping of iPSC-CMs can be performed with CalTrack, identifying subtle changes that are critical to understanding pathogenic signals. Existing strategies with these capabilities typically require expensive hardware and skilled manual interventions (Table I in the Data Supplement). Additionally, to allow automated, high-throughput assessment of calcium transient changes for therapeutic screening, we aimed for efficient data acquisition and analyses. CalTrack is freely available using a MatLab pipeline or provided as a compiled version for users without MatLab access.

CalTrack defines calcium transients in primary cardiomyocytes from animal models^6^ and iPSC-CMs, including cells on patterned substrates^13,14^ or in engineered 3-dimensional tissue systems^15^ (Figure 1 and Figure I and II in the Data Supplement). The algorithm automates background subtraction, performs photobleach correction, uses masks to identify individual cells in image stacks with multiple cells per field of view, averages all identified transients, and performs transient fitting with parametric output. This code relies only upon providing a directory of video files (.avi,.vsi,.mov,.czi,.m4v,.mp4, and others) that can be converted to .tif stacks or previously extracted fluorescence data in an excel file (Figure 2). CalTrack is user-friendly (User Manual in the Data Supplement) and computationally inexpensive so that it can be run on a local computer. On average, the algorithm requires 140 seconds to convert 10 video files (250 frames) to .tif stacks and 55 seconds to analyze and export computed parameters into excel files. In comparison, manual conversion of similar videos (120 seconds), extraction of calcium traces (200 seconds), and computation of the parameters is considerably slower. In addition, manual computation of average representative traces, with baseline correction can impair standardization. We show that CalTrack’s rapid automated analysis and fitting of hundreds of cells on a local computer provide robust statistical power for analyzing changes in cellular calcium transients in comparison to a commercial software. As CalTrack only requires fluorescence microscopy, or extracted traces, to rapidly analyze large data sets, we suggest that this platform provides a critical resource for the cardiac biology community to accelerate experimentation in physiology, pathology, and therapeutic screening.

**Figure 1.**
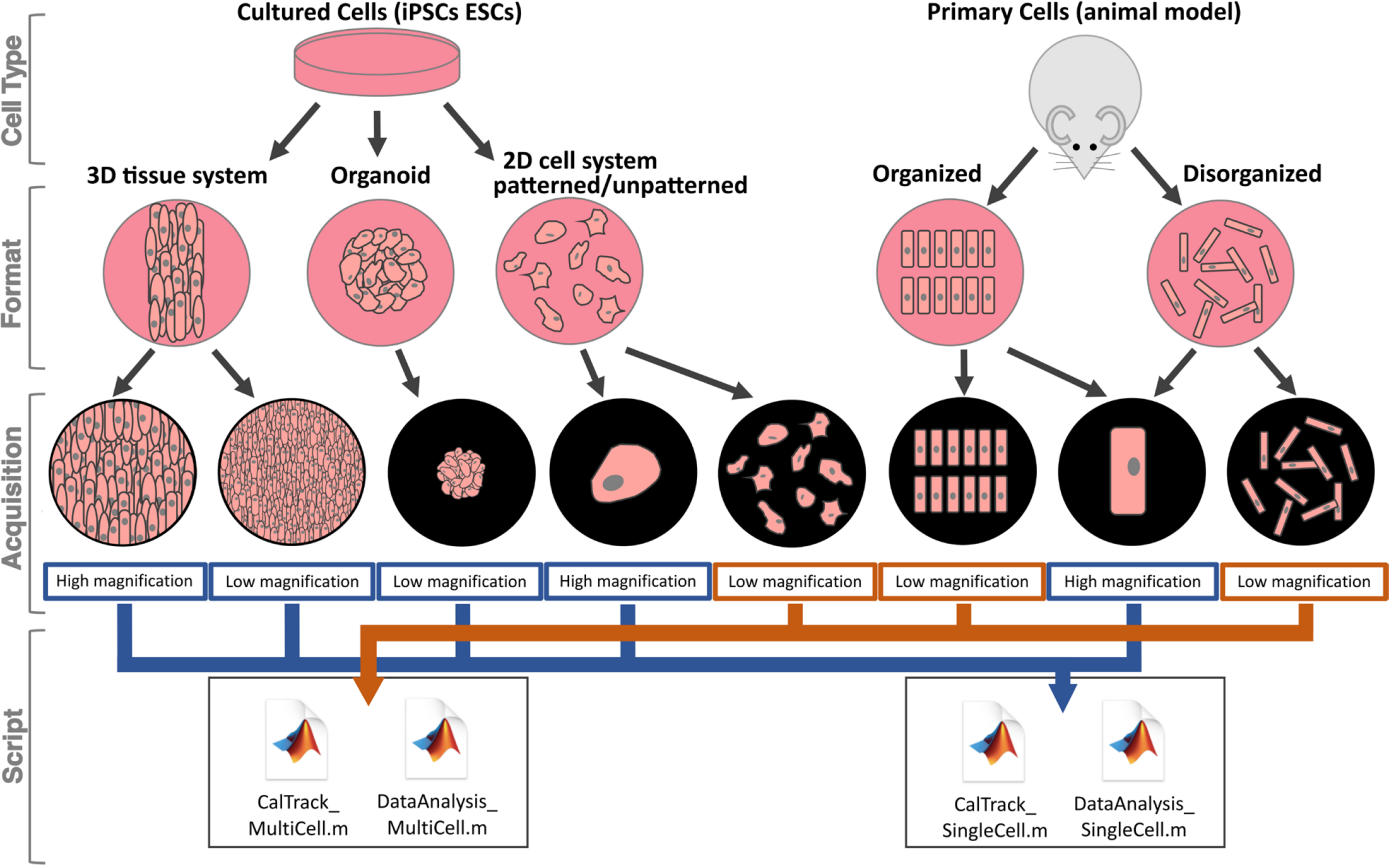
**The variety of cardiovascular model systems and constructs that can be automatedly analyzed using CalTrack.** CalTrack can be applied to multiple cellular systems, both organized and disorganized primary cardiomyocytes from animal models and immortalized cell types (induced pluripotent stem cell–derived cardiomyocytes [iPSC-CMs] and embryonic stem cell derived cardiomyocytes [ESC-CMs]), using formats that include 3-dimensional (3D) tissue systems, organoids, and 2-dimensional (2D) cell culture systems.

**Figure 2.**
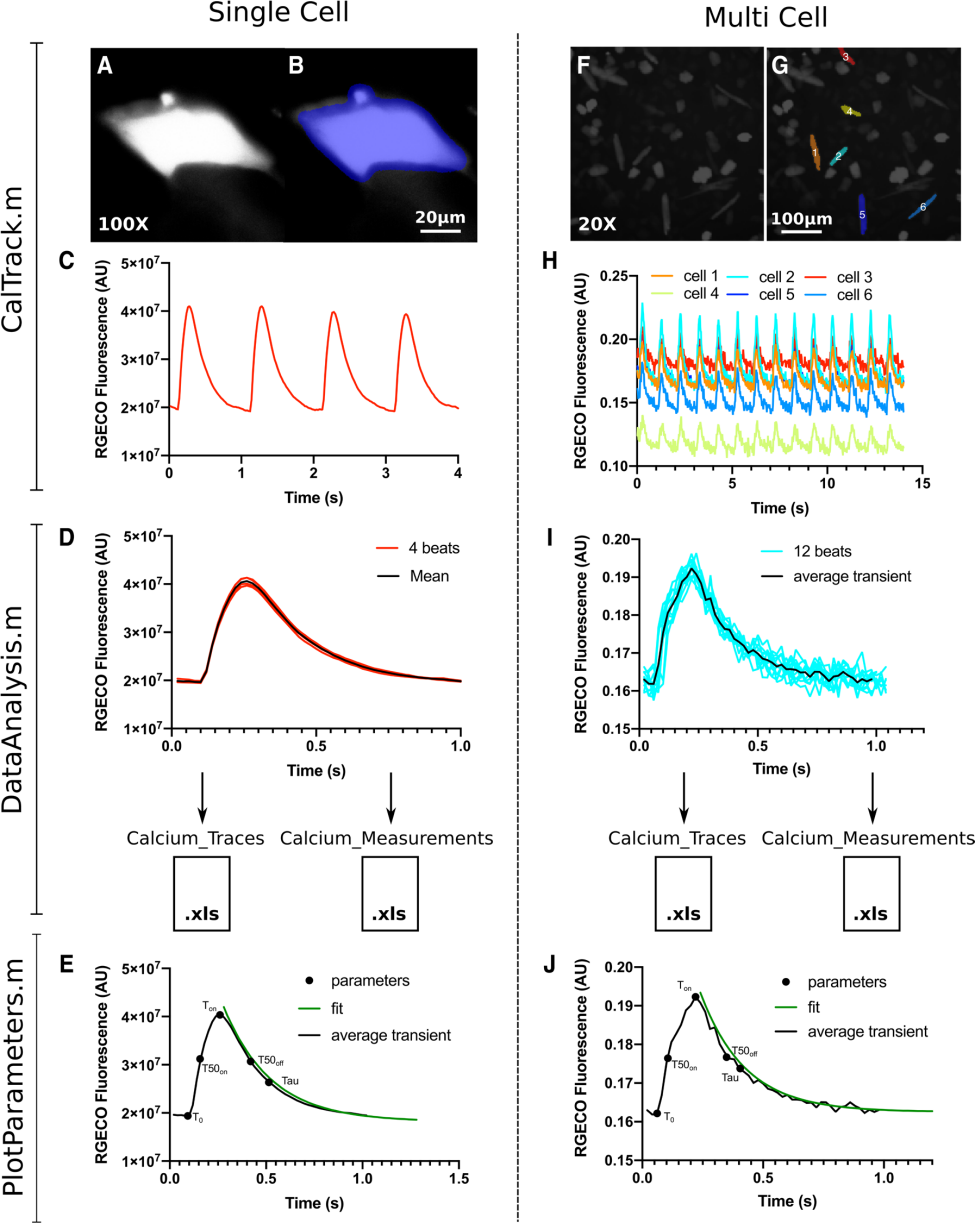
**The CalTrack calcium analysis pipeline can be used for Single cells or multicell image stacks.** **A**, A single induced pluripotent stem cell–derived cardiomyocytes (iPSC-CM) imaged using an ×100 objective that has been masked (**B**) by CalTrack for segmentation and fluorescent trace extraction. **C**, From the masked iPSC-CM CalTrack extracts a raw calcium trace. **D**, CalTrack then automatedly segments the beats (red lines) and can average them into one trace (black line). **E**, CalTrack can then provide all parameters from each individual transient or from the mean transient of a single cell. **F**, The same analysis routine can be applied to low magnification (×20) image stacks with multiple cells per field of view. **G**, The CalTrack segmentation mask identifies single cells for transient analysis and provides raw fluorescent traces for each cell (**H**), which can then be individually segmented and averaged (**I**) for automatic parametric output (**J**). AU indicates arbitrary units.

## Methods

### Data Availability

The CalTrack code and trial data sets are openly available and maintained on GitHub at the following URL: https://github.com/ToepferLab/CalTrack.

### Mathematical Basis of CalTrack Calcium Transient Analysis

To extract calcium traces, CalTrack relies on the Bio-Formats toolbox^16^ for MatLab (Mathworks, Inc, Natwick, MA). CalTrack initially reads all frames in the video stack and averages the value of all pixels to generate a mask. Where multiple cell segmentation is required, the average image is subjected to a difference of Gaussians filter^17^ to increase contrast in local boundaries. Contrast-limited adaptive histogram equalization and erosion-dilation enhance this to produce a segmented cell mask. This is applied to extract the raw fluorescence within each cell per frame, as the sum of all pixels in each cell (n):


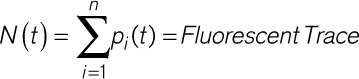


where 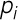 represents each pixel intensity.

Below we provide a simplified mathematical version (see Data Supplement for additional details) of the process of extraction and analysis of traces that have successfully passed quality control and have either been corrected for photobleach or have been deemed by the user not to require photobleach correction. Previously extracted traces can also be processed with CalTrack.

First, to select the beginning of each fluorescent transient and perform transient segmentation, the entire trace is converted to an array of the difference of each intensity value with its previous value:


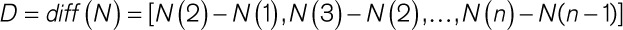


The onsets of fluorescence increase, which correspond to the start of calcium transients, match the peak values of D. Peaks in D (termed D_p_) are selected as onsets of calcium-driven fluorescent transients by thresholding to a minimum prominence of 50%. The beginning of each transient is selected by subtracting an offset of 10% of the cycle length to D_p_ (eg, 100 ms for 1 Hz pacing), in the original trace. Individual transients are cropped from the whole fluorescent trace by specifying their beginning and ending points at D_p_-offset and D_p+1_-offset, respectively. Next, the transients are averaged to a single fluorescence intensity transient unless the option of measuring parameters on each individual transient is selected by the user, in which case the following is performed on every transient. When measuring parameters on each individual transient, a measurement of the SD of all individual transients’ parameters is provided as a quality control metric.

As an alternative to automatic transient segmentation, CalTrack offers the user the option to perform this process by defining the beginning of the first calcium transient via event markers such as the time of electrical pacing, if known.

To characterize all temporal parameters of the obtained average transient, the baseline intensity must be determined. This is achieved by averaging the last points of the trace that correspond to a temporal window of 20% of the cycle length (eg, 200 ms for 1 Hz pacing). Subsequently, the peak of the trace is selected as the maximum intensity value and the transient magnitude is calculated as max–baseline. Peak fluorescence/baseline fluorescence (F_max_/F_0_) is calculated as the peak value divided by the baseline. Next, since fluorescent traces may have a lower baseline at the end of the trace than at the beginning, a more robust value for the baseline is defined as baseline+(0.03×magnitude). This enables automated definition of the beginning and end of each transient in the fluorescent trace and is the smallest change that can support automation while minimally altering the measured temporal parameters (Figure III in the Data Supplement). The redefined baseline value is used to calculate all temporal parameters via linear interpolation (ie, interpolation of time at intensity values corresponding to 0%, 10%, 50% and 90% of the magnitude, which has been recalculated with the redefined baseline), with the exception of tau (and its fitting parameters), which is calculated by fitting the decaying arm of the fluorescent trace with an exponential decay curve with equation 
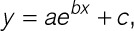
 and calculating tau as 
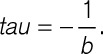
 The goodness-of-fit value is also calculated and reported for quality control. For traces without uniform electrical pacing (ie, more fluorescent transients are identified than the manually-input pacing frequency justifies) or traces that display aberrations reminiscent of irregular behavior, such as early or delayed afterdepolarizations (Figure IV in the Data Supplement), additional information is reported as output. This includes the beat-to-beat time as the average time interval between peaks, the number of intervals used to compute the beat-to-beat time, and the cell and beat number where early or delayed afterdepolarizations occurred. These traces may later undergo post hoc processing. Here an automated analysis of the regular events is carried out (ie, excluding the irregular parts of the trace), and reported measurements include all of the above in addition to the number of total and irregular beats, as well as classifying irregular beats as an early or delayed afterdepolarization.

Finally, for quality control, the signal-to-noise ratio is calculated for the extracted traces as the average of the transient values (above baseline) divided by the SD of values below the baseline.

### Production of Synthetic Calcium Transients for CalTrack Benchmarking

Synthetic calcium transients were simulated using a biophysically detailed electro-mechanical model^18^ of adult human ventricular myocyte obtained through the coupling of the electrophysiology ToR-ORd^19^ and the contractility Land^20^ models. Simulations were conducted in MatLab, using the numerical solver *ode15s* with solutions reported every 1 ms, corresponding to an acquisition frequency of 1000 frames per second (FPS). The model was stimulated at 0.5, 1, and 2 Hz for 200 beats, delivering a stimulus current of −53 µA/µF for 1 ms. The last 5 beats of each simulation were saved. For each pacing frequency, twenty 5-beat traces were generated by scaling either the L-type calcium or the rapid delayed rectifier potassium currents’ conductance. Calcium traces were downsampled to match experimental acquisition frequencies. Noise equivalent to 2.5× the SD of the final 300 ms of each calcium trace was added to each trace. Resemblance to commonly acquired experimental data was confirmed on all traces visually before analyzing with CalTrack.

### Generation of WT and TNNI3^R21C/+^ Missense iPSC-CMs for Calcium Transient Analyses

A heterozygous pathogenic missense variant *TNNI3*^R21C^ that causes hypertrophic cardiomyopathy^21^ was introduced using CRISPR/Cas-9 technology as previously described and in Methods in the Data Supplement^10,22–24^ (please see the Major Resources Table in the Data Supplement). Targeted iPSC subclones were sequenced to confirm the *TNNI3*^R21C/+^ genotype (Figure V in the Data Supplement), differentiated into iPSC-CMs via Wnt pathway modulation, and plated in a 2-dimensional assay plate as described in detail in the Supplemental Methods and identically as previously described.^25^ Cells were treated with dimethylsulfoxide (DMSO) or Mavacamten 0.3 to 3 µmol/L as described in detail in Methods in the Data Supplement.

### Guinea Pig Cardiomyocyte Isolation and Manipulation

Guinea pig studies were performed with protocols that were reviewed and approved by the Animal Welfare and Ethical Review Board at the University of Oxford and conform to the UK Animals (Scientific Procedures) Act, 1986. Adult left ventricular cardiomyocytes (adolescent male, 10–15 weeks in age) were isolated and immediately processed for ratiometric calcium analyses as previously described^26^ (see Methods and Major Resources Table in the Data Supplement). Paced Fura2-loaded cells were studied using the Ionoptix (Waltham, MA) platform IonWizard, according to manufacturer’s protocols, and by CalTrack (see Results). Alternatively, isolated cardiomyocytes were co-transduced for 48 hours with adenovirus carrying a red genetically encoded calcium indicator (RGECO)^6^ and a cDNA encoding either WT *TnnI3* or *TnnI3* R145G, and cultured in plates for imaging.^6^ Videos of 0.5 Hz electrically paced cardiomyocytes at 37 °C were acquired at 25 FPS. Pharmacological effects were assessed in guinea pig cardiomyocytes that have been pretreated with either DMSO or 10 µmol/L levosimendan for 15 minutes at 37 °C before imaging.

### Statistical Analysis

All individuals performing data analysis were blinded to the treatment group under study. Postanalysis processing was unblinded. Statistical analyses were performed using GraphPad Prism version 8.4.2 for macOS (GraphPad Software, San Diego, CA, www.graphpad.com). Normality in all data sets, defined as alpha <0.05, was assessed by the D’Agostino-Pearson normality test. Single comparisons of data modeled by a normal distribution was assessed by a double-tailed Student *t* test; multiple comparisons use a 1-way ANOVA with post hoc correction for the number of comparisons. Data that were not modeled by a normal distribution was assessed by a nonparametric Mann-Whitney test. When multiple comparisons were tested in unpaired data, a Kruskal-Wallis was used with post hoc Dunn correction. For multiple comparisons in paired data (Figure 4, Figures III and VII in the Data Supplement) where the normality test was not satisfied, data were tested with a nonparametric Friedman test with Dunn multiple comparisons correction. In all instances, a significance cutoff of *P*<0.05 was used.

**Figure 3.**
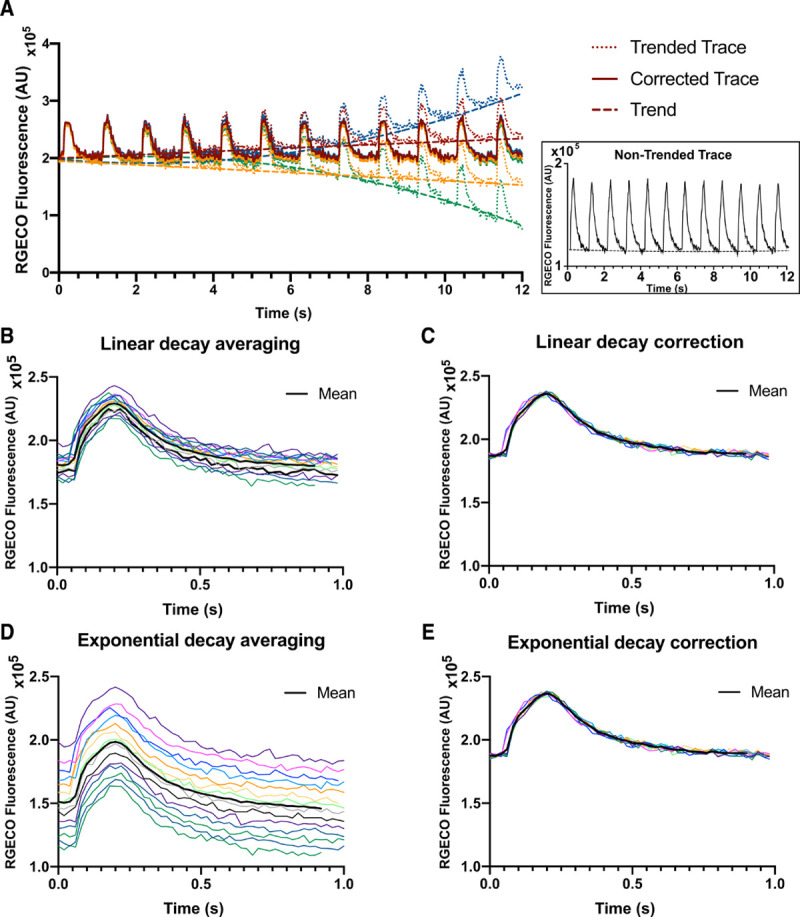
**CalTrack successfully corrects linear and exponential photobleach events.** **A**, Simulated linear and exponential baseline shifts are applied to a fluorescent calcium trace (inset). Colored trend lines are fit to the baseline shifted data by CalTrack. These are used to correct shifts in baseline. **B** and **D**, Segmented individual transients from traces that need baseline correction due to baseline drift caused by linear decay (**B**) or exponential decay (**D**). CalTrack correction of baseline drifts functions robustly for linear (**C**) or exponential (**E**) baseline shift. AU indicates arbitrary units.

## Results

### CalTrack Pipeline Overview and Applications

CalTrack analyzes image stacks/videos acquired with fluorescent calcium reporters in iPSC-CMs or isolated adult cardiomyocytes (Figure 1). Alternatively, extracted traces (collected in excel files) that bypass the need to derive data from stacks/videos can also be analyzed. After incubation with chemical calcium dyes, such as Fura2, Fluo-4,^2^ or following transduction with viruses carrying calcium probes (eg, RGECOs/GCaMPs^27^), cells are imaged using a fluorescent microscope at a range of magnifications (typically ×100–×10) to provide image stacks of either single or multiple cells per field of view (Figure 2). Image acquisition rates should at minimum be 25 FPS but ideally should be 40 to 100 FPS to allow the accurate capture of calcium transients. However, data acquired at up to 1000 data points per second can be analyzed (Figure VI in the Data Supplement). CalTrack applies a mask to these image stacks to identify either single or multiple cells in a field of view (Figure I in the Data Supplement). In the case of imaging single iPSC-CMs at ×100 or equivalent optical magnification, CalTrack identifies the area that the cell occupies in the field of view and applies a fixed mask to the cell to measure fluorophore intensity in each frame of the acquisition (Figure 2A and 2B). Fluorophore intensity is, therefore, measured within the cell’s boundary at maximum relaxation throughout each frame of the acquisition (Figure 2A through 2C and Figure IA in the Data Supplement). The individual transients can then be segmented and aligned to create a mean transient per cell (Figure 2D), which is used to determine the parameters that define the calcium transient. These include the decay constant tau (Tau), determined by curve fitting to the mean transient, time to reach calcium peak (T_on_), time to 90% of peak (T90_on_), time to 50% of peak (T50_on_), time to 10% of peak (T10_on_), F_max_/F_0_, time for calcium transient decay (T_off_), time of 10% decay (T10_off_), time of 50% calcium decay (T50_off_), time of 90% decay (T90_off_), calcium transient duration (CD), and signal-to-noise ratio for the calcium trace. Examples of the CalTrack fitting and parametric determination are illustrated in Figure 2E and in the CalTrack user guide.

For low magnification acquisition where multiple cells are within a single field of view, CalTrack can distinguish cells by applying a mask that defines cells within a field of view (Figure 2F and 2G and Figure I in the Data Supplement). This allows the simultaneous fluorescence intensity assessment of many cells from one image stack (Figure 2H). Each of the identified cells can then be individually segmented so that a mean transient per cell can be computed (Figure 2I). The computed mean is then used for fitting and measurement of transient parameters (Figure 2J).

### CalTrack Robustly Detects Cardiomyocytes in Low Magnification Image Stacks

Imaging calcium transients in multiple cells within a single field of view at low magnification can improve throughput. To enable the analysis of this data, CalTrack constructs a cell mask to identify individual cells and excludes rounded dead cells by filtering out nonelongated cells. This strategy yielded a 1.36% false-negative rate and 4.04% false-positive rate (Figure IB through ID in the Data Supplement). Cells detected by CalTrack are presented to the user for final quality control, and the end-user can further exclude cells as false positives. As the predominance of false positives reflect overlapping cells within high plating densities (Figure IE in the Data Supplement), cultures should aim to minimize individual cellular overlap to reduce false-positive detection of cells.

### CalTrack Corrects for Linear and Exponential Baseline Drift in Calcium Transient Acquisitions

Baseline drift in fluorescent microscopy with calcium indicators can be caused by multiple events including photobleaching, loss of fluorophore from the cell, or cell damage. CalTrack enables the investigator to select whether elimination from processing or correction of baseline drift is desirable. To correct baseline drift, a second-degree polynomial, applicable for both linear and exponential drifts, is used to determine the baseline trend; this is subsequently subtracted from the original trace to detrend the data (Figure 3A). Data that lacks baseline drift remains unaffected (Figure 3A, inset). Before baseline correction, linear baseline drift would skew mean calcium transient segmentation (Figure 3B). Correction of the linear baseline drift reduces individual transient drift and provides a robust corrected mean transient (Figure 3C). The same applies to an exponential decay in baseline, which can be successfully modeled and removed from datasets with this approach (Figure 3D and 3E).

#### CalTrack Provides Analysis of Irregular Calcium Transients Including Early Afterdepolarizations and Delayed Afterdepolarizations

To account for irregular fluorescent transients, which cannot be analyzed as part of the main automated analysis, CalTrack has additional post hoc analysis capabilities. These analyses require high signal-to-noise ratio in the extracted traces to allow robust detection of irregular transients. CalTrack classifies irregular traces as nonadherence to pacing or calcium spikes reminiscent of early and delayed afterdepolarizations (Figure IV in the Data Supplement). CalTrack can exclude the irregular events from the multibeat trace and measure parameters from the regular transients as it would usually in adherent transients (Figure 2). The irregular transients are subsequently analyzed and CalTrack provides a count of the number of total transients, the number of excluded events, and the number of irregular events, including designation of events as early or delayed afterdepolarizations.

#### Assessment of Quantitative Calcium Concentrations Using CalTrack

We harnessed CalTrack to quantify intracellular calcium using the ratiometric indicator Fura2 in WT adult guinea pig cardiomyocytes (Figure VI in the Data Supplement). When running CalTrack the user can either provide a calibration curve for the ratiometric indicator or alternatively, a calibration curve is generated within CalTrack by inputting the fluorescence intensity values at known calcium concentrations for a specific ratiometric indicator. This calibration is then used with the fluorescence ratio of the ratiometric indicator to generate absolute raw and smoothed calcium concentration traces, demarcating T_0_, providing profiles with temporal parameters from baseline and peak calcium concentrations, and quantifying the baseline and peak concentration of intracellular calcium (Figure VI in the Data Supplement).

#### Benchmarking of CalTrack With Synthetic Simulated Calcium Transient Data

**Figure 4.**
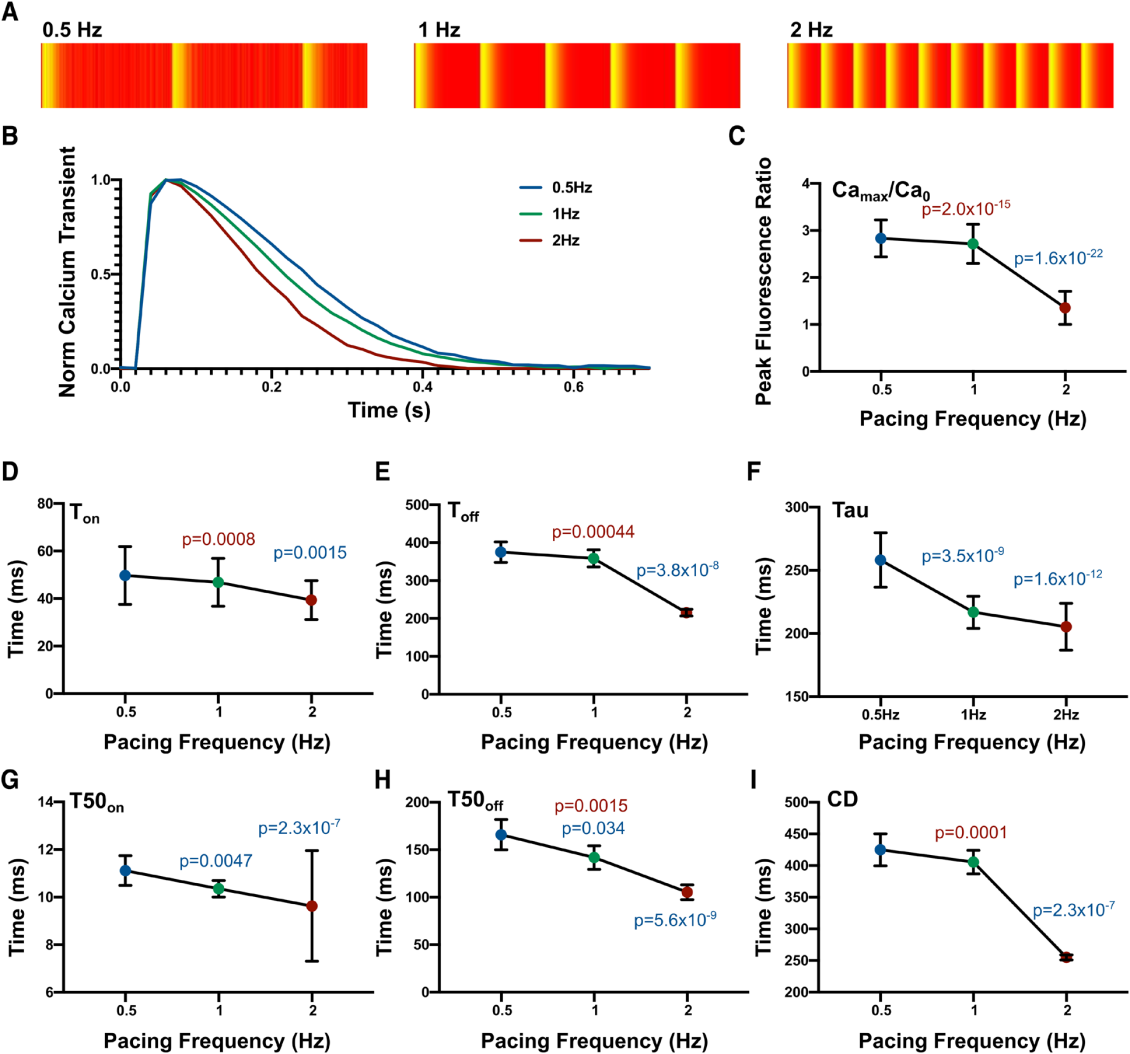
**CalTrack analyzes computationally simulated calcium transients at multiple beating frequencies with fidelity.** **A**, 0.5–2 Hz Kymographs produced by CalTrack. **B**, Average normalized calcium transients (n=20 simulated traces for each pacing frequency). **C**, Increases in pacing frequency decrease normalized peak calcium concentration (equivalent to peak fluorescence/baseline fluorescence [F_max_/F_0_]). **D–I**, Increasing pacing frequency significantly accelerates all calcium transient properties assessed by CalTrack (time to reach calcium peak [T_on_], time for calcium transient decay [T_off_], calcium decay constant (Tau), time to 50% of calcium peak [T50_on_], time of 50% calcium decay [T50_off_], and calcium transient duration [CD]). Data presented as mean and SD, colors of data points represent 0.5 Hz pacing (blue), 1 Hz pacing (green), and 2 Hz pacing (red). For Ca_max_/Ca_0_ and Tau statistical analysis is performed by 1-way ANOVA with a post hoc Sidak correction. T_on_, T_off_, T50_on_, T50_off_, and CD were tested with a paired nonparametric test Friedman test with Dunn multiple comparisons correction, 3 comparisons. In both instances, a *P*<0.05 was defined as the significance cutoff. Ca_max_/Ca_0_ indicate maximal calcium/baseline calcium.

CalTrack has been designed to function for multiple pacing frequencies, allowing the user to define the experimental pacing frequency. To test the fidelity of the underlying CalTrack code, we used simulated calcium transients obtained from 20 virtually generated human cardiomyocytes and several pacing frequencies from 0.5 Hz to 2 Hz (Figure 4). Variation of electrical pacing frequency affects simulated calcium transient parameters as expected (Figure 4A through 4I). When using CalTrack to analyze these data, the mean over 20 simulated calcium transients shows marked changes in calcium transient characteristics with pacing frequency (Figure 4B). Normalized calcium peak (equivalent to F_max_/F_0_ in experiments) decreases with increasing pacing frequency (Figure 4C). T_on_ and T_off_ are faster at higher pacing frequencies (Figure 4D and 4E). The calcium transient decay constant, tau, decreased with increasing pacing frequencies (Figure 4F). T50_on_, T50_off_, and CD were accelerated as pacing frequencies increased (Figure 4G through 4I).

To determine the interference of noise with measurements, random noise was added to simulated calcium traces at 1 Hz pacing (Figure VII in the Data Supplement). Measurements of peak normalized calcium, tau, time to 50%, peak calcium, time to 50%, and complete decay were unchanged at signal-to-noise ratio 15 to 70. However, the time to 50% peak calcium was increased (Figure VIIH in the Data Supplement), and time to complete decay was decreased (Figure VIIG in the Data Supplement) at low signal-to-noise ratio of 10. Therefore, data acquired below signal-to-noise ratio of 15 may be unreliable and should be closely scrutinized by the end-user.

#### Quantitative Calcium Analyses in Adult TnnI3^R145G/+^ Cardiomyocytes With Benchmarking Comparison Between Automated CalTrack Analysis and Manual, User-Driven IonWizard Analysis

**Figure 5.**
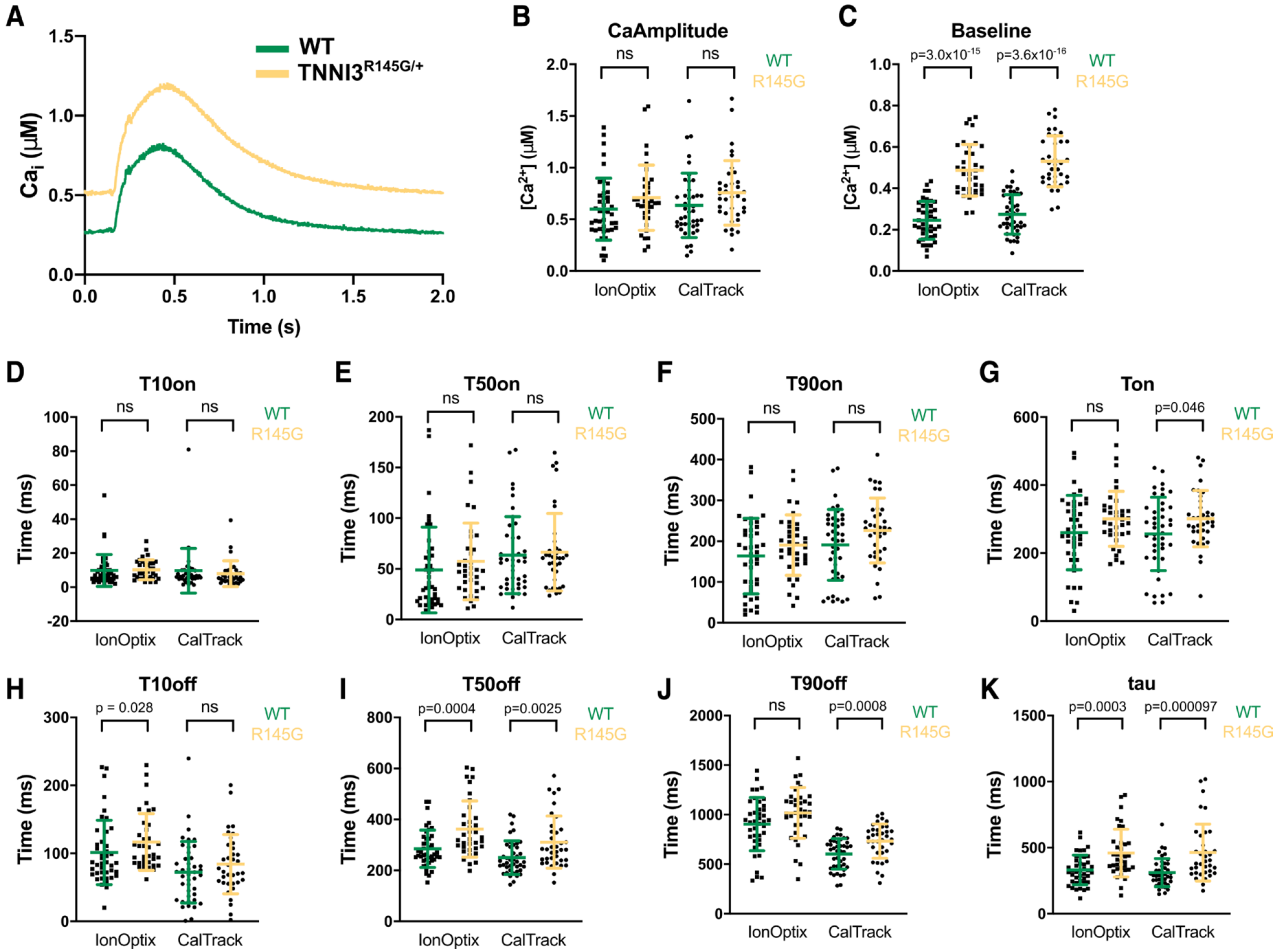
**Automated CalTrack quantification of calcium concentration and kinetic parameters in guinea pig wild-type (WT) and *TnnI3*^*R145G/*+^ cardiomyocytes is comparable to manual IonOptix IonWizard software analysis.** **A**, Average calcium transients are identical by analysis via IonWizard or CalTrack analysis. Average traces are made of n=42 WT and n=36 for *TnnI3*^*R145G/*+^ cells. **B**, Calcium amplitude is unaffected by *Tnni3*^*R145G/*+^. **C**, Baseline [Ca^2+^] is increased in *TnnI3*^*R145G/*+^ cells. Time to 10% of peak (T10_on_; **D**), time to 50% of calcium peak (T50_on_; **E**), and time to 90% of peak (T90_on_; **F**) are unaffected by *TnnI3*^*R145G/*+^. **G**, time to reach calcium peak (T_on_) is significantly longer in *TnnI3*^*R145G/*+^ cardiomyocytes when assessed by CalTrack analysis. **H**, Time of 10% decay (T10_off_) is unaffected by *TnnI3^R145G/+^*. **I**, Time of 50% calcium decay (T50_off_) is longer in *TnnI3*^*R145G/*+^ cardiomyocytes. **J**, Time of 90% decay (T90_off_) is assessed to be longer in *TnnI3*^*R145G/*+^ cardiomyocytes by CalTrack analysis. **K**, Calcium decay constant (Tau) is significantly longer in *TnnI3*^*R145G/*+^ cardiomyocytes. Statistical analysis is performed by a 2-tailed Student *t* test (Baseline, T_on_, T90_on_, and T90_off_) or by nonparametric Mann-Whitney test (CaAmplitude, T10_on_, T50_on_, T10_off_, T50_off_, tau) with a significance cutoff of *P*<0.05. No statistical comparisons are made between IonOptix and CalTrack. Ns indicates nonsignificance.

We compared ratiometric calcium data from adult guinea pig cardiomyocytes, which had either been transfected with human TnnI3^+/+9^ or with the R145G variant troponin I (*TnnI3*^*R145G/*+^),^6,28^ which had been loaded with Fura2 (Figure 5). Both algorithms detected significantly increased baseline and peak calcium concentrations without altering calcium amplitudes (Figure 5A through 5C). The times to 10%, 50%, and 90% of peak calcium were not significantly different, although CalTrack detected a modest increase in the time to peak calcium (Figure 5D through 5G). Both software detected abnormalities in calcium decay times, although not identically and prolonged tau in the *TnnI3*^*R145G/*+^ versus WT cardiomyocytes (Figure 5H through 5K). Although these analyses demonstrated comparable data acquisition and interpretation by both software platforms, the overall operator times for using IonWizard was 20-fold longer on average per cell analyzed (CalTrack ≈1 minute, IonWizard ≈20 minutes).

#### CalTrack Detects Calcium Transient Responses to Pharmacological Agents, Levosimendan and Isoproterenol, in Adult Cardiomyocytes

**Figure 6.**
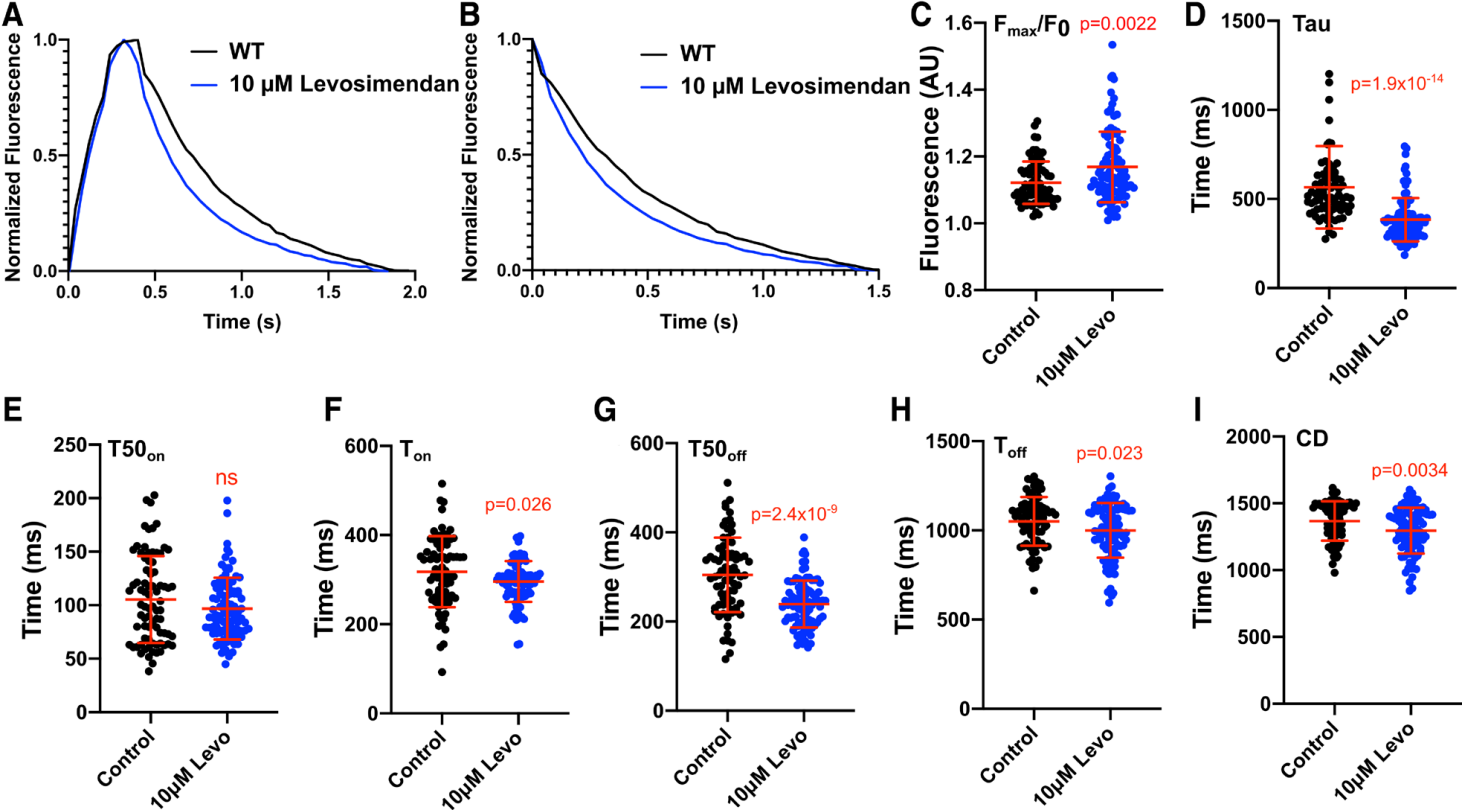
**CalTrack defines calcium transient changes caused by the application of levosimendan in paced (0.5 Hz) adult guinea pig cardiomyocytes.** **A**, Normalized mean calcium transients and mean calcium decays (**B**) from control (n=79) or levosimendan (Levo) 10 µmol/L (n=96) treated guinea pig cardiomyocytes. Levo treatment increased peak fluorescence/baseline fluorescence (F_max_/F_0_; **C**), decreased calcium decay constant (Tau; **D**), and did not affect time to 50% of calcium peak (T50_on_; **E**). Levo treatment shortened time to reach calcium peak (T_on_; **F**), time of 50% calcium decay (T50_off_; **G**), time for calcium transient decay (T_off_; **H**). and calcium transient duration (CD; **I**). Data presented as mean and SD. Statistical analysis is performed by a 2-tailed Student *t* test (CD, T_off_, T50_off_) or by nonparametric Mann-Whitney test (F_max_/F_0_, Tau, T50_on_, T_on_) with a significance cutoff of *P*<0.05. AU indicates arbitrary units; and WT, wild-type.

We studied WT adult guinea pig cardiomyocytes at baseline (n=79) and after treatment (n=96) with 10 μM of the calcium sensitizer levosimendan.^29^ Normalized mean calcium transients from 0.5 Hz paced cells (Figure 6A) and calcium decays (Figure 6B) that were obtained after levosimendan treatment increased F_max_/F_0_ (*P*=0.0022; Figure 6C) and reduced tau (*P*=1.9×10^-14^; Figure 6D). T50_on_ was not statistically significantly altered by levosimendan (Figure 6E), but T_on_ was shortened (*P*=0.026; Figure 6F). Levosimendan accelerated T50_off_ (*P*=2.4×10^-9^; Figure 6G) and T_off_ (*P*=0.023; Figure 6H) and reduced CD (*P*=0.0034; Figure 6I). Drug-induced changes in fluorescence identified by CalTrack are consistent with prior studies in guinea pig cardiomyocytes^6^ and pharmacological activities of levosimendan. Levosimendan binds troponin C and increases calcium affinity, which underlies its positive inotropic effects,^30,31^ and also potently inhibits phosphodiesterase activity,^32^ thereby augmenting PKA (protein kinase A) and cAMP effects that increase cellular relaxation rates^33^ and promote lusitropy.

CalTrack also detected the effects of the β-adrenergic agonist isoproterenol on 1 Hz paced WT iPSC-CMs (Figure VIII in the Data Supplement). In comparison to untreated cells, isoproterenol increased calcium kinetics, showing both a more rapid increase in normalized calcium fluorescence and significantly reduced tau, T_on_, and T50_off_, consistent with previously reported data.^34^

#### CalTrack Detects Abnormal Calcium Transients in Guinea Pig Cardiomyocytes Expressing the TnnI3^R145G/+^ HCM Variant

**Figure 7.**
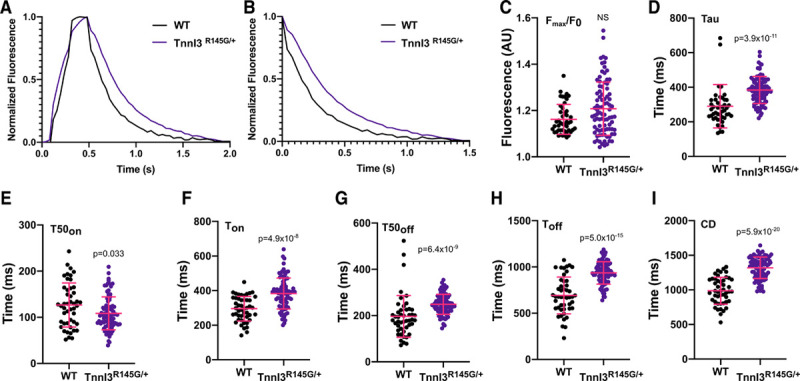
**CalTrack defines calcium transient changes caused by the *TnnI3*^*R145G/*+^ hypertrophic cardiomyopathy (HCM) variant in paced (0.5 Hz) adult guinea pig cardiomyocytes.** **A**, Normalized mean calcium transients and calcium decays (**B**) from wild-type (WT; n=47) or *TnnI3*^*R145G/*+^ (n=88) guinea pig cardiomyocytes. The *TnnI3*^*R145G/*+^ variant does not affect peak fluorescence/baseline fluorescence (F_max_/F_0_; **C**), and calcium decay constant (Tau; **D**). Time to 50% of calcium peak (T50_on_) is accelerated (**E**), but time to reach calcium peak (T_on_; **F**), time of 50% calcium decay (T50_off_; **G**), time for calcium transient decay (T_off_; **H**), and calcium transient duration (CD; **I**) are all slowed in comparison to WT. Data presented as mean and SD. Statistical analysis is performed by a 2-tailed Student *t* test (T_on_, T_off_, and CD) or by nonparametric Mann-Whitney test (F_max_/F_0_, Tau, T50_on_, and T50_off_) with a significance cutoff of *P*<0.05. ns indicates nonsignificant. AU indicates arbitrary units.

We compared calcium homeostasis in 0.5 Hz paced adult WT (n=47) and *TnnI3*^*R145G/*+^ (n=88) guinea pig cardiomyocytes (see Methods in the Data Supplement). In comparison to WT cardiomyocytes, the average calcium transients (Figure 7A) and decay traces (Figure 7B) indicated that *TnnI3*^*R145G/*+^ did not significantly alter F_max_/F_0_ (Figure 7C) but significantly prolonged tau (*P*=3.9×10^-11^) and caused faster T50_on_ (*P*=0.033; Figure 7E). T_on_ was slowed in *TnnI3*^*R145G/*+^ guinea pig cardiomyocytes (versus WT, *P*=4.9×10^-8^; Figure 7F). T50_off_ (*P*=6.4×10^-9^; Figure 7G) and T_off_ (*P*=5.0×10^-15^; Figure 7H) were longer in *TnnI3*^*R145G/*+^ guinea pig cardiomyocytes and CD was prolonged (*P*=5.9×10^-20^; Figure 7I).

#### CalTrack Detects Basal and Drug-Induced Calcium Changes in iPSC-CMs With an Endogenous TNNI3^R21C/+^ HCM Variant

We used CalTrack to assess the calcium transients from isogenic iPSC-CMs with and without an endogenous heterozygous pathogenic troponin I variant (*TNNI3*^*R21C/*+^) that causes HCM^21^ (see Methods in the Data Supplement). A founder *TNNI3*^*R21C/*+^ mutation in South Lebanon causes malignant HCM with sudden cardiac death, which often precedes hypertrophy,^35^ likely due to abnormal calcium handling from disruption of PKA-mediated phosphorylation of its N-terminal molecular switch.^21,36^

**Figure 8.**
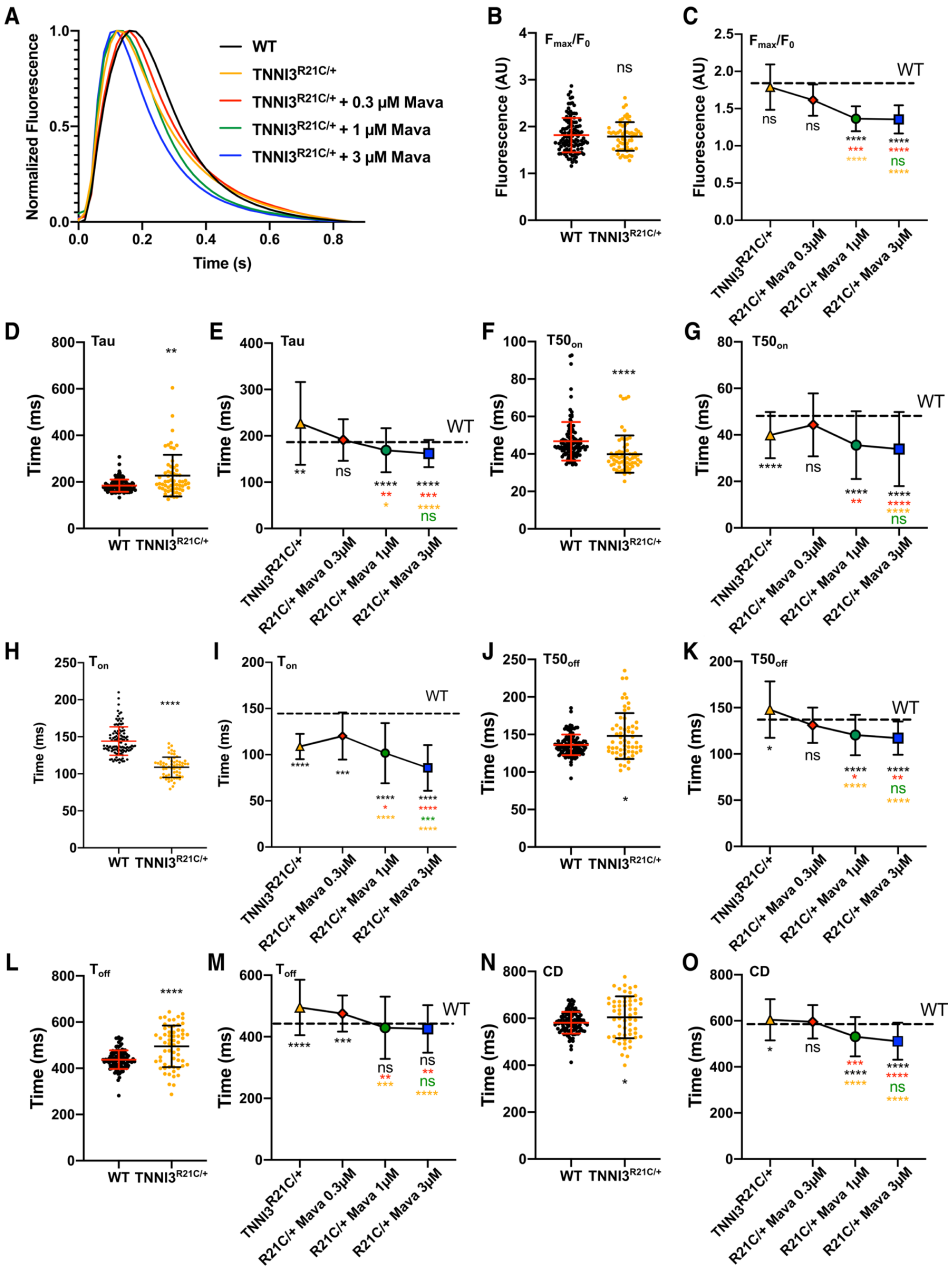
**CalTrack defines calcium phenotypes and drug responses in paced induced pluripotent stem cell–derived cardiomyocytes (iPSC-CMs) harboring the hypertrophic cardiomyopathy (HCM) *TNNI3*^*R21C/*+^ variant.** **A**, Averaged calcium transient traces of wild-type (WT; n=113 and *TNNI3*^*R21C/*+^ (n=60) cardiomyocytes with 0.3 μM (n=40), 1 μM (n=59), and 3 μM mavacamten (n=92). **B**, *TNNI3*^*R21C/*+^ did not alter peak fluorescence/baseline fluorescence (F_max_/F_0_). **C**, Mavacamten reduces F_max_/F_0_ in a dose-dependent manner. **D** and **E**, Calcium decay constant (Tau) is prolonged in *TNNI3*^*R21C/*+^ cardiomyocytes and shortened by increasing mavacamtem dose. **F**, *TNNI3*^*R21C/*+^ shortens time to 50% of calcium peak (T50_on_). **G**, Mavacamten has variable, dose-dependent effects on T50_on_ in the *TNNI3*^*R21C/*+^ cardiomyocytes. **H** and **I**, time to reach calcium peak (T_on_) is shortened in *TNNI3*^*R21C/*+^ cardiomyocytes at baseline (**H**) and further decreased by Mavacamten (**I**). **J** and **K**, Time of 50% calcium decay (T50_off_) is prolonged in *TNNI3*^*R21C/*+^ cardiomyocytes (**J**) and shortened (**K**) with increasing doses of mavacamten. **L** and **M**, time for calcium transient decay (T_off_) is prolonged in *TNNI3*^*R21C/*+^ cardiomyocytes (**L**) and shortened (**M**) with increasing mavacamten dose. **N** and **O**, Calcium transient duration (CD) is statistically significantly prolonged in *TNNI3*^*R21C/*+^ cardiomyocytes (**N**) and reduced (**O**) with increasing doses of mavacamten. Data presented as mean and SD. Statistical analysis is performed by nonparametric Kruskal-Wallis with post hoc Dunn correction for multiple comparisons (9 comparisons). Significances are denoted on each panel where *P<0.05, **P<0.01, ***P<0.005, ****P<0.0001. Color of significance denotation defines the treatment group compared. Please see Table III in the Data Supplement for exact *P* values. AU indicates arbitrary units; and Mava, Mavacamten.

We studied 1 Hz paced cells treated with and without mavacamten,^37^ an allosteric myosin ATPase inhibitor,^38^ which improves relaxation deficits associated with HCM thick filament variants^39–42^ and also influences calcium via unknown mechanisms.^6^ Using CalTrack outputs for the mean calcium transient in each cell analyzed (excel file), we defined an average transient at baseline and for each perturbation (Figure 8). Mutant iPSC-CMs had increased T_50off_, T_off_, and tau at baseline, which is consistent with previous work in a knock-in mouse model carrying the R21C variant^43^ that exhibits marked diastolic insufficiency and slowed myofilament relaxation.

*TNNI3*^*R21C/*+^ iPSC-CMs were not statistically different from WT iPSC-CMs when assaying F_max_/F_0_ at baseline (Figure 8B), whereas mavacamten further reduced F_max_/F_0_ at each dose (Figure 8C, Table III in the Data Supplement). Other calcium parameters that were abnormal in *TNNI3*^*R21C/*+^ were reversed by treatment with 0.3 μM mavacamten, including prolonged tau (versus WT, *P*=0.001, Figure 8D; normalized in Figure 8E) and significantly faster T50_on_ (versus WT, *P*=3.0×10^-8^; Figure 8F; normalized in Figure 8G). *TNNI3*^*R21C/*+^ also had faster T_on_ (vs. WT, *P*= 2.2×10^-16^, Figure 8H) and dose-dependently accelerated by mavacamten (Figure 8I), whereas prolonged T50_off_ (vs. WT, *P*=0.044; Figure 8J) was normalized (Figure 8K). T_off_ was longer (*P*=1.7×10^-5^; Figure 8L) and dose-dependently corrected by mavacamten (Figure 8M). CD was longer in *TNNI3*^*R21C/*+^ iPSC-CMs when compared with WT (*P*=0.035, Figure 8N) but was dose-dependently shortened by mavacamten (Figure 8O). CalTrack identified that 8% of *TNNI3*^*R21C/*+^ iPSC-CMs displayed delayed afterdepolarizations (n=60), in comparison to 0.9% in WT iPSC-CMs (n=113).

The effects of increasing mavacamten dose on WT iPSC-CMs are shown in Figure IX in the Data Supplement. F_max_/F_0_ was increased by 3 µmol/L mavacamten treatment. T50_on_ and T_on_ were dose-dependently reduced by mavacamten treatment (Figure IXA through IXC in the Data Supplement). T50_off_ decreased at 1 µmol/L and 3 µmol/L doses (*P*=0.023 and *P*=0.0045, respectively; Figure IXD in the Data Supplement). At 3 µmol/L mavacamten, there was accelerated decay in Tau (*P*=0.0018; Figure IXE in the Data Supplement), an accelerated T_off_ *P*=0.024; Figure IXE in the Data Supplement), and decreased CD (*P*=0.000019; Figure IXG in the Data Supplement).

## Discussion

We demonstrate that CalTrack, a high-throughput automated analysis pipeline that capitalizes on advanced imaging capacities and acquisition of high frame rates by fluorescent microscopy, provides robust assessment of calcium transient in cultured or patterned cardiomyocytes, and cardiomyocytes within engineered tissues. This extends to quantitative ratiometric analysis of calcium concentrations within cells when data is acquired at high temporal resolution (>1000 data points per second). Harnessing rapid screening technologies to visualize and image multiple cells in single fields of view, CalTrack acquires and automatedly and rapidly analyzes data in high throughput.

Quantitative assessment of calcium transients by CalTrack can be used in computational investigations into the pathogenic mechanisms underlying cardiovascular conditions and their modulation under therapeutic interventions.^44,45^ Computational modeling and simulation of cardiac pathophysiology can then be used to augment experimental datasets provided by CalTrack to enable a mechanism-driven understanding of cellular phenotypes,^44,45^ thereby providing a platform for testing hypotheses in human cardiomyocytes as alternatives to animal models^46^ so as to characterize physiological and pathological processes at baseline and in response to interventions.

Contemporary open-source software for analyzing calcium transients focuses on neuronal cell types^47–51^ and characterizes irregular traces generated by neurons. Although these platforms have some utility in cardiomyocytes, they are cumbersome for use in this system, and cannot run automatedly. These issues limit high volume acquisition of cellular calcium transients and require significantly longer analysis times. Some laboratories overcome these issue through custom scripts, which typically lack automation, have variable parameter definitions, or limited parameter extraction precluding cross-study comparisons.^52–56^ By contrast, software automation eliminates manual user input and associated user error/bias so as to provide calcium transient data in a defined, standardized way that can be compared between cells and experimental conditions.

Although CalTrack is easily deployable for analysis across a variety of cardiac cells within a range of experimental systems, the platform has limitations. Analytical accuracy is compromised at low acquisition rates because of the rapid kinetics of the calcium transients generated in cardiomyocytes. For example, acquisition at 25 FPS will sample data every 40 ms, resulting in a theoretical temporal resolution of 92 ms (based on the Nyquist theorem, whereby resolution is equal to 1/2.3 of the sampling frequency). Hence the starting point of the calcium transient may be resolved as much as 40 ms further from whence it commences. This effect increases at lower sampling rates and is independent of the software used for analysis. Thus, although CalTrack uses interpolation to measure parameters smaller than the acquisition window, the acquisition frequency dictates accuracy, especially in parameters related to the start and fast phase of the transient. We suggest that this factor underlies the measurement difference of T50_on_ in WT human iPSC-CMs (≈50 ms, acquired at 50 FPS) compared with WT guinea pig cardiomyocytes (≈100 ms, acquired at 25 FPS). Nevertheless, CalTrack enables comparative analyses for lower frame rate data, where our findings using CalTrack are complementary to previous findings generated using the same experimental data set that had been analyzed with manual user-driven analysis and previously reported.^6^ Additionally, we note that individual transient registration may be perturbed with extremely high frame rate data, as over-sampling reduces the prominence of point-to-point differences, from which the start of a calcium transient is defined. However, CalTrack data acquired at 1000 FPS is downsampled to 100 FPS solely to identify the start of the calcium transients; the raw 1000 FPS data is then used for parametric analyses. Therefore, high-frequency data acquired by line scanning or by the use of a photomultiplier tube can also be robustly and rapidly analyzed by CalTrack. As with many platforms, high data noise may limit the accuracy of the algorithm in fitting key parameters, but this is largely overcome by the trace averaging used in CalTrack. Indeed, almost identical analysis fidelity of values was obtained with CalTrack and the IonOptix IonWizard software (Figure 5). When needed, CalTrack also provides the end-user the ability to check fitting fidelity for all steps of the analysis process so that data quality control standards can be identified. Notably, CalTrack can also be used to automatedly analyze data obtained using a ratiometric calcium indicator, such as Fura2.

We demonstrate the utility of CalTrack to define perturbations in components of calcium homeostasis in adult guinea pig cardiomyocytes and human iPSC-CMs. We show that levosimendan, an agent with ionotropic effects by sensitizing troponin C^57^ and a potent phosphodiesterase inhibitor^32^ that stabilizes PKA, increased F_max_/F_0_, accelerated calcium decay, and accelerated time to peak calcium, which manifested as shorter CDs in guinea pig cardiomyocytes similar to human myocardium.^33^ iPSC-CMs treated with the β-adrenergic agonist isoproterenol activated calcium transients, due to PKA-dependent increases in L-type calcium channel activity,^58^ release of sarcoplasmic reticulum calcium by phosphorylation of phospholamban,^59^ and ryanodine receptor activity.^60^

CalTrack robustly detected differences in mutated sarcomeric thin filament proteins. Two pathogenic HCM variants in *TNNI3* both had prolonged tau and longer T_off_ times. CalTrack also identified an 8-fold higher frequency of delayed afterdepolarizations in TNNI3^R21C/+^ iPSC-CMs when compared with WT iPSC-CMs. These findings result in decreased cellular relaxation, as assessed by SarcTrack in TNNI3^R21C/+^ iPSC-CMs, which often manifest as diastolic abnormalities in HCM hearts,^6,26^ and may promote afterdepolarizations and arrhythmias that contribute to sudden cardiac death.^35^ The *TnnI3*^*R145G/*+^ variant had a longer T_on_, whereas the *TNNI3*^*R21C/*+^ variant had a shorter T_on_ when compared to respective WT cells. These findings are significant and are mirrored in the respective contractility profile of each variant^26^ (Figure X in the Data Supplement). Notably, these data also show that the allosteric myosin ATPase modulator mavacamten, which corrects the contractile abnormalities in HCM thick filament variants, normalized tau and T_off_ in *TNNI3*^*R21C/*+^ iPSC-CMs albeit with a dose-dependent reduced F_max_/F_0_, time to peak, and CD (Figure IX in the Data Supplement). As such, mavacamten treatment may salvage some of the diastolic insufficiency caused by HCM mutations that are concomitant with increased lengths of calcium decays. These findings support recent studies,^61^ suggesting that mavacamten influences calcium cycling with potential therapeutic efficacy in addition to its established effects on myosin ATPase.^38,61^

Finally, we note that the combined use of endogenous genetically encoded GFP (green fluorescent protein) tag on titin^22^ concurrent with the red spectrum RGECO calcium fluorophore allows concurrent imaging of both sarcomere contraction and calcium transients in cardiomyocytes (Figure X in the Data Supplement). Dual functional assessments with a cell-permeant fluorophore represent the potential for future development and combination of SarcTrack with CalTrack, so as to enable wide-spread analyses using calcium-sarcomeric shortening loops (Figure XIB in the Data Supplement).

In summation, CalTrack has demonstrated applicability as a rapid automated calcium transient analysis platform for a wide variety of cardiomyocyte constructs and sources. CalTrack provides calcium amplitude and temporal parameters from single or multiple adult or iPSC-derived cardiomyocytes within a field of view and can analyze previously extracted data from proprietary equipment. The algorithm provides high-fidelity measurements, limited by the quality of data input (eg, acquisition rate of image stacks and noise), and is easily adopted and applied on local computers with rapid automated data processing rates. We expect this open-source tool will advance assessments of calcium transients across multiple experimental cardiovascular fields.

## Acknowledgments

We thank the members of the Image and Data Analysis Core at Harvard Medical School (HMS) for their discussions and technical assistance with programming CalTrack.

## Sources of Funding

Support for this study was provided in part by the Wellcome Trust and British Heart Foundation Centre of Research Excellence (BHF-CRE) Oxford (C.N. Toepfer: Sir Henry Wellcome fellowship 206466/Z/17/Z and BHF-CRE Intermediate Transition fellowship RE/18/3/34214), Y. Psaras: the BHF-CRE RE/18/3/34214, G.G. Repetti: the Sarnoff Foundation, British Heart Foundation (RG/12/16/29939), and British Heart Foundation Centre of Research Excellence (Oxford; to H. Watkins and C.S. Redwood). Additional funding for A.J. Sparrow, P. Robinson, and C.S. Redwood: PG/18/68/33883 and P. Robinson and H. Watkins: CH/1992001/6764. B. Rodriguez holds a Wellcome Trust Senior Research Fellowship (214290/Z/18/Z) and an NC3Rs (National Centre for the Replacement, Refinement and Reduction of Animals in Research) Infrastructure for Impact Award (NC/P001076/1). A. Bueno-Orovio is supported by BHF Intermediate Basic Science Fellowship to A. Bueno-Orovio (FS/17/22/32644). The Fondation Leducq (J.G. Seidman and C.E. Seidman) the Engineering Research Centers Program of the National Science Foundation (J.G. Seidman, and C.E Seidman: National Science Foundation (NSF) Cooperative Agreement number EEC-1647837), the National Institutes of Health (J.G. Seidman and C.E. Seidman: 5R01HL080494 and 5R01HL084553), and the Howard Hughes Medical Institute (C.E. Seidman). F. Margara is funded by the Personalised In-Silico Cardiology project, European Union’s Horizon 2020 Research, and innovation programme under the Marie Sklodowska-Curie grant agreement 764738.

## Disclosures

None.

## Supplemental Materials

CalTrack User Guide

Expanded Materials and Methods

Data Supplement Figures I–XI

Data Supplement Tables I–III

Data Supplement Movies I–III

## Supplementary Material



## Novelty and Significance

### What Is Known?

- Measurements of calcium transients in cardiomyocytes provide insights into homeostasis and pathophysiologic processes and define drug effects.
- Tools that automatedly segment cells, extract calcium transients, and output data in high throughput are not available.
