# Supplementary figures and images for: CalTrack: High-Throughput Automated Calcium Transient Analysis in Cardiomyocytes
Source: Circ Res. 2021 May 21;129(2):326–41. doi: 10.1161/CIRCRESAHA.121.318868 (PMC8260473; doi:10.1161/CIRCRESAHA.121.318868)

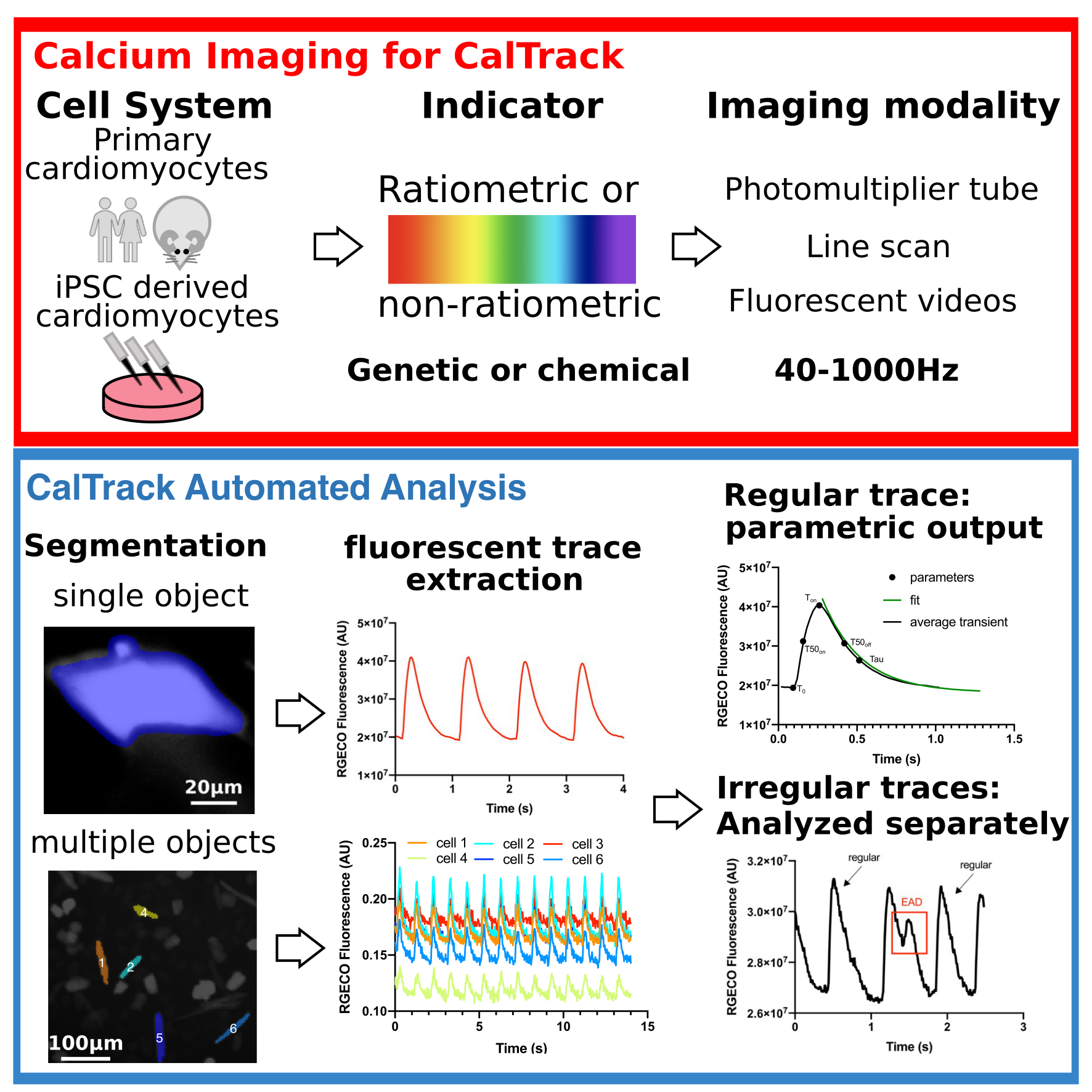

Supplement: Supplementary file 4 [file res-129-326-s004.jpg]
