# Supplementary material for: CalTrack: High-Throughput Automated Calcium Transient Analysis in Cardiomyocytes
Source: Circ Res. 2021 May 21;129(2):326–41. doi: 10.1161/CIRCRESAHA.121.318868 (PMC8260473; doi:10.1161/CIRCRESAHA.121.318868)
Supplement: Supplementary file 5 [file res-129-326-s005.pdf]

Major Resources Table

In order to allow validation and replication of experiments, all essential research materials listed in the Methods should be included in the Major Resources Table below. Authors are encouraged to use public repositories for protocols, data, code, and other materials and provide persistent identifiers and/or links to repositories when available. Authors may add or delete rows as needed.

Animals (in vivo studies)

Species	Vendor or Source	Background Strain	Sex	Persistent ID / URL
Guinea Pig	Marshall BioResources	Albino Pirbright	Male	

Genetically Modified Animals

	Species	Vendor or Source	Background Strain	Other Information	Persistent ID / URL
**Parent - Male**					
**Parent - Female**					

Antibodies

Target antigen	Vendor or Source	Catalog #	Working concentration	Lot # (preferred but not required)	Persistent ID / URL

DNA/cDNA Clones

Clone Name	Sequence	Source / Repository	Persistent ID / URL

Cultured Cells

Name	Vendor or Source	Sex (F, M, or unknown)	Persistent ID / URL
WT	PGP1	M	
TNNI3R21C/+	Seidman Lab	M	

Data & Code Availability

Description	Source / Repository	Persistent ID / URL
CalTrack	github	https://github.com/ToepferLab/CalTrack

Other

Description	Source / Repository	Persistent ID / URL

DOI [to be added]
