# Supplementary material for: CalTrack: High-Throughput Automated Calcium Transient Analysis in Cardiomyocytes
Source: Circ Res. 2021 May 21;129(2):326–41. doi: 10.1161/CIRCRESAHA.121.318868 (PMC8260473; doi:10.1161/CIRCRESAHA.121.318868)
Supplement: Supplementary file 6 [file res-129-326-s006.pdf]

ONLINE METHODS

Generation of the TNNI3$^{R21C/+}$ missense iPSC line (and accompanying control) for calcium transient acquisition

The heterozygous HCM variant *TNNI3*$^{R21C/+}$ was created using CRISPR/Cas9 technology by introducing a missense mutation into the *TNNI3* allele in human iPSCs harboring GFP-tagged titin.[22] Our approach was to design a single-stranded guide RNA sequence (gRNA) that serves as CRISPR RNA (crRNA).[23] Our genomic region of interest in the *TNNI3* gene served as the target of the double-stranded DNA breaks (DSB) induced by Cas9, a necessary step to facilitate homology directed repair (HDR) with a template engineered to replace the targeted nucleotide with the desired missense mutation. First, using the bioinformatics online program Benchling, a gRNA (5'-AAGCGCGGTAGTTGGAGGAG-3') was identified in close proximity to the target site. Adjacent to our site-specific guide was the sequence 5'-CGG-3', designating the protospacer-adjacent motif (PAM) site, which serves as a recruiter for Cas9 and has the format NGG, such that the complete guide RNA target sequence is 5'-N19-NGG-3'. An asymmetric HDR template (5'-TCTTGGATCCCTCCGGCGCCTGTACTCTGCCCCCAGGAAGCCCCGTCCCACCTTGGCG TGCGGCTCCGTGGCATAAGCGCGGTAGTTGGAGGAGCGACATCTGATTGGGGCTGGT GCAGGGCGAGGTT-3') was designed that would replace the C at p677 with a T, changing the amino acid arginine (<u>C</u>GC) to cysteine (<u>U/T</u>GC). The asymmetry of the HDR arms is intended to increase efficiency of sequence replacement[10]. We performed genome editing using a Cas9 ribonucleoprotein protein transfection protocol. First a ribonucleoprotein (RNP) complex was created by incubating tracrRNA (Integrated DNA Technologies) with the gRNA for 5 minutes at 95 **°C,** then 10 minutes at 70 °C, then 5 minutes at 45 **°C**[23]. Cas9 nuclease (Alt-R HiFi, Integrated DNA Technologies) was then added and incubated at room temperature for 40-60 minutes. This RNP complex was then combined with the HDR template and added to iPSCs, which were then electroporated using a nucleofector (Lonza) and plated. iPSC clones were picked and resulting *TNNI3*$^{R21C/+}$ sequences were confirmed using sanger sequencing and MiSeq **(See Online Figure V)**.

Generation of iPSC-CMs

Monolayer differentiation of control and WT cell lines was performed via Wnt pathway modulation with small molecule inhibitors. Cells were induced to the mesodermal layer with 12

μM CHIR99021 in RPMI1640 / N21 minus insulin for 24 hours. This was considered to be day 0 of differentiation. At the end of 24 hours, media were replaced with RPMI1640 / N21 minus insulin. On day 3, cells were induced to cardiac lineage specification with 5 μM IWP2 in conditioned media (equal volumes of old and fresh RPMI1640 / N21 minus insulin) for 48 hours. Cells were then cultured in the absence of insulin to day 10. Spontaneous contraction was observed on day 9-11 of differentiation. Cells were subjected to metabolic selection for 48 hours starting on day 11, with glucose-free RPMI / N21 plus insulin. Cells were then cultured to day 26 in RPMI1640 / N21 plus insulin.

Plating and viral transduction of iPSC-CMs with RGECO

On day 27, iPSC-CMs were dissociated with 10x TrypLE enzyme for 5 minutes. The dissociation reagent was quenched with plating media (20% fetal bovine serum, 10 μM Thioglycerol, 1% penicillin/streptomycin in RPMI 1460). Cells were collected in suspension, centrifuged at 200 rcf for 3 minutes and cells were resuspended in plating media. Cells were transduced in suspension with adenovirus carrying RGECO (MOI = 8) and plated at a density of 1.5×10^5 on Geltrex-coated glass bottom 35 mm plates, to be imaged three days later.

Small molecule treatments on iPSC-CMs

Plates were prepared in triplicate per small molecule treatment and concentration. Cells were incubated for ten minutes with the myosin inhibitor, mavacamten, and the β-adrenergic stimulator Isoproterenol at 0.3, 1 and 3 μM prior to imaging. Controls were provided by triplicate plate of equivalent concentrations of dimethylsulfoxide (DMSO), as well as a triplicate of plates incubated in the absence of any treatment to capture the mutant phenotype. This was repeated in three plates of wild-type cardiomyocytes, differentiated as above from. A minimum of 30 cells were recorded per plate.

Mavacamten treated iPSC-CMs

Plates were prepared in triplicate per small molecule concentration. iPSC-CMs were plated on day 15-20 and kept in 2D culture until contractility was observed, but no later than day 27 from differentiation. On day 27, cells were transduced with adenovirus carrying RGECO at higher

multiplicity of infection (MOI = 40) than applied to all other treatments, and imaged on day 30 and 31.

Guinea Pig cardiomyocyte isolation

This investigation was approved by the Animal Welfare and Ethical Review Board at the University of Oxford and conforms to the UK Animals (Scientific Procedures) Act, 1986, incorporating Directive 2010/63/EU of the European Parliament. Adult left ventricular cardiomyocytes were isolated from guinea pig (male, 400g) hearts by collagenase perfusion as previously described[6]. Left ventricular cardiomyocytes (1.5 x 10^5 cells per ml) were incubated in $ACCITT_3$ culture medium at 37°C and 5% CO_2. Immediately after isolation, cardiomyocytes were adenovirally co-transduced for 48 hours with either RGECO and WT cTnI, or RGECO and cTnI R145G to give expression of WT cTnI and cTnI R145G at an approximate 50% level compared to endogenous troponin as previously implemented and described[6]. Cardiomyocytes from each adenovirally co-transduced group were imaged on an Olympus IX81 inverted microscope (Olympus, Japan) with a C-9100-13 EMCCD camera (Hamamatsu, Japan). Videos of 0.5 Hz electrically paced cardiomyocytes at 37°C were acquired at 25 fps (560/25 nm excitation, 620/60 nm emission with a 565 nm dichroic mirror). For assessing the effect of Levosimendan on guinea pig cardiomyocytes, cells were treated with either DMSO or 10 µM levosimendan for 15 minutes at 37°C then imaged as described.

Signal to noise ratio simulation

For this, synthetic calcium transients were used as described in the main text. The model was stimulated at 1 Hz and noise equivalent to 2.5 times the standard deviation of the final 300 ms of each calcium trace was added to each trace. The SNR was calculated on these initial traces and noise was enhanced to achieve SNR at 10,15, 20, 25, 30, 35, 40, 45, 50, 55, 60, 65, 70. Synthetic calcium traces were stored in MatLab structures and analyzed with the CalTrack algorithm for all parameters.

ONLINE FIGURES I – XI

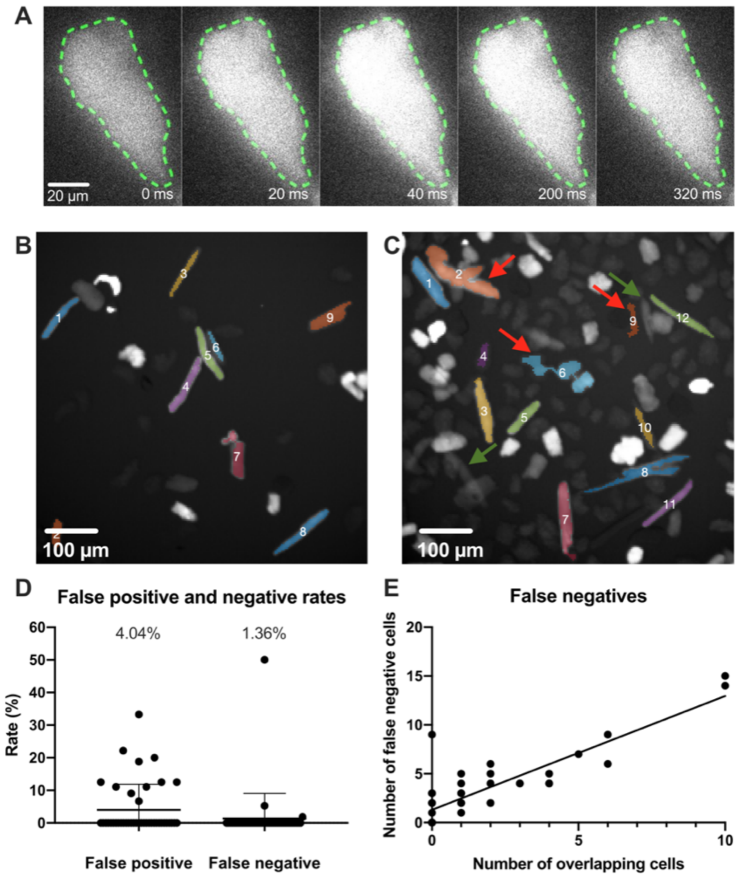

Online Figure I: Application of fixed-size mask and cardiomyocyte detection for multiple-cell videos. A) Cell masks generated to a fixed size (green interrupted line) applied over cardiomyocytes at a fixed location that encompasses the entire cell throughout the image stack, regardless of intensity or potential motion of the cell. Sequential images selected to show the upstroke and decay of a calcium transient. Representative image shown only for high magnification, as motion artefacts are not appreciable at low magnification. **B)** and **C)** Representative examples of masking for multiple primary guinea pig cardiomyocytes (x 20 magnification) at two densities illustrate more robust selection of cells at low cell density. Red arrows – false positive, green arrows – false negative. **D)** False detection rates (n = 42 videos). **E)** Loss of data represented by false negatives (n = 22) is shown to increase with increasing number of overlapping cells due to high cell density ($p = 0.064 \times 10^{-10}$), tested with simple linear regression with statistical significance cut-off of $p<0.05$.

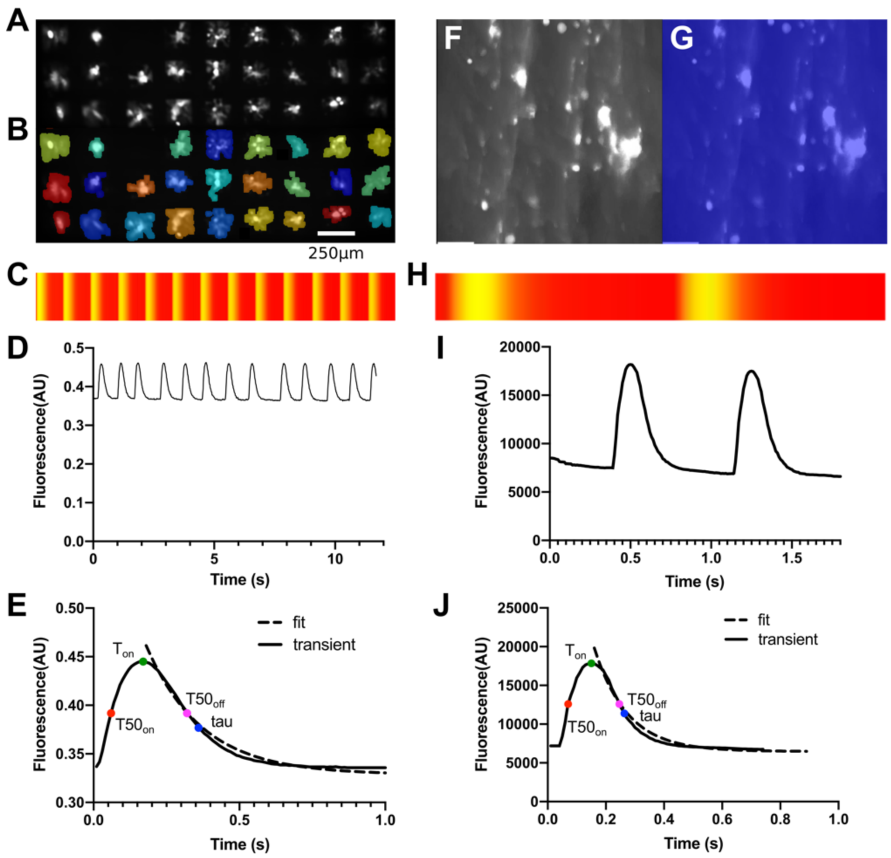

Online Figure II: CalTrack analysis of multiple patterned iPSC-CMs or single iPSC derived Engineered Heart Tissues (EHTs). A) Single frame of multiple iPSC-CMs on patterned cell islets from Werley et.al.[14] **B)** The same frame as in A with a CalTrack-generated mask to identify regions of interest for calcium transient assessment. **C)** Representative kymograph of a single cell for 12 seconds of the acquired video stack corresponding to a single islet from images in A and B. **D)** Fluorescent transient profile measured over 12 seconds by CalTrack. **E)** Mean calcium transient produced by CalTrack with transient parameters marked on the plot. (Mean and SEM for all cells measured can be found in Online Table II). **F)** Single frame of an EHT from a movie published in Saleem et.al.[15]**G)** CalTrack-generated mask applied to the entire field of view to measure the calcium transient from the single EHT in the field of view (mask represented in varying hues of blue; note that it spans the entire image). **H)** CalTrack-rendered kymograph from the short EHT calcium transient movie stack. **I)** CalTrack measured calcium transient fluorescent profile from two beats of the EHT. **J)** Mean calcium transient produced by CalTrack from the two beats observed in I. Parameters are marked on the plot and results from the fit can be found in Online Table II.

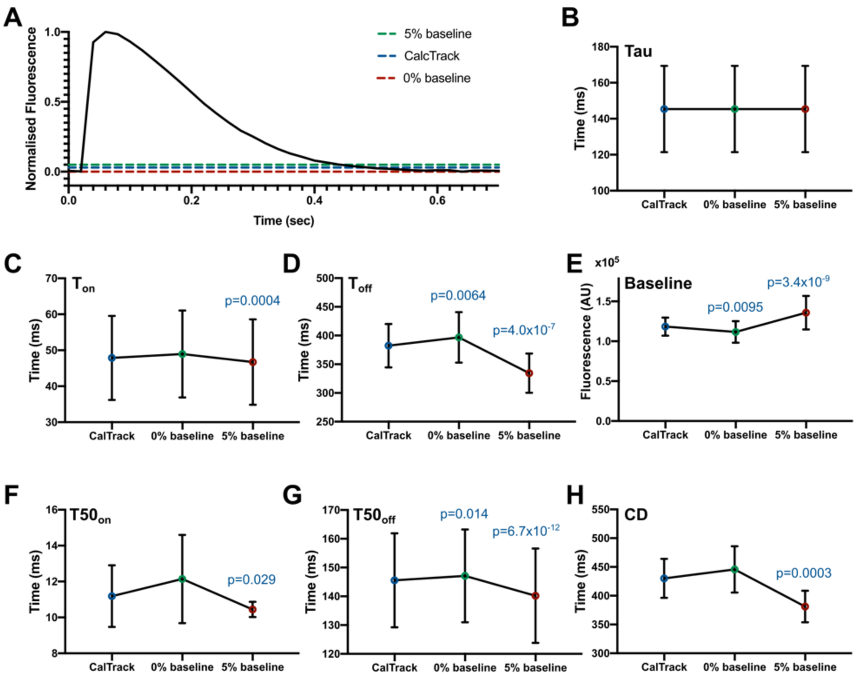

Online Figure III: Effect of altering baseline fluorescence estimation 5% or CalTrack (3%) in comparison to hand-picked data. **A)** Fluorescent calcium transient simulated you from a computer model of cardiomyocyte calcium flux. Baseline fluorescence is denoted by hand measurement (0% baseline, n = 20), a 5% automated baseline can be applied (5% baseline, n = 20), and CalTrack analysis uses an intermediate 3% baseline (n = 20) to increase fidelity of automated baseline fitting in noisy traces. **B**) Tau measurement, showing no appreciable change in Tau using the three different baseline methods. **C)** T_{on} is significantly altered by using a 5% baseline ($p < 0.01$) but not by the CalTrack baseline picking. **D)** CalTrack T_{off} is significantly different by pairwise analysis from a 0% hand-picked or 5% automated baseline. **E)** CalTrack analysis of absolute fluorescence baseline is significantly higher than a hand-picked 0% baseline and lower than a 5% automated baseline. **F)** $T50_{on}$ as measured by CalTrack is shorter than by a hand-picked baseline, but indistinguishable from a 5% automated baseline. **G)** $T50_{off}$ measured by CalTrack is shorter than by a 0% hand-picked baseline and longer than the 5% baseline. **H)** CD is shorter for CalTrack measurement than for hand-picked 0% baselines, but longer than the 5% automated baseline. Baseline, Tau, T_{off}, and $T50_{off}$ were analyzed by one-way ANOVA with a post-hoc Sidak correction with a significance cut-off of $p<0.05$. T_{on}, $T50_{on}$, CD were assessed using a Friedman test with Dunn's multiple comparisons correction. In both instances a significance cut-off of $p<0.05$ was used

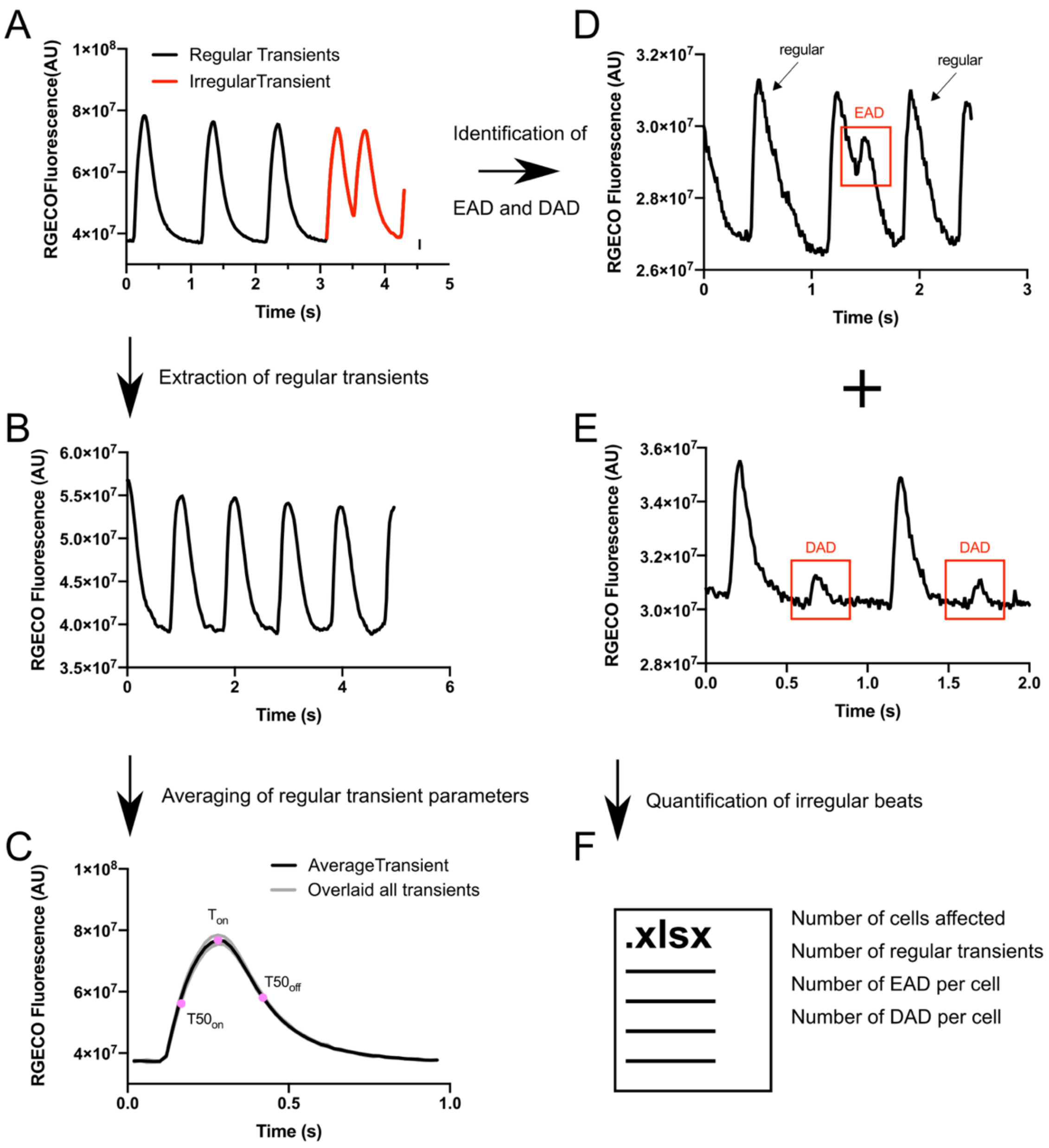

Online Figure IV: Post processing for irregular calcium traces. A) Representative example of irregular trace. **B)** CalTrack separates the regular traces from the entire transient. **C)** Regular traces are averaged, and all parameters are measured as in the standard pipeline. Irregular traces are separated from the regular ones and recorded as EADs if a second peak is detected before a transient reaches baseline **(D)**, or DADs if a smaller peak is detected after the transient reaches baseline **(E)**. **F)** All data is collated and exported in Excel spreadsheet format.

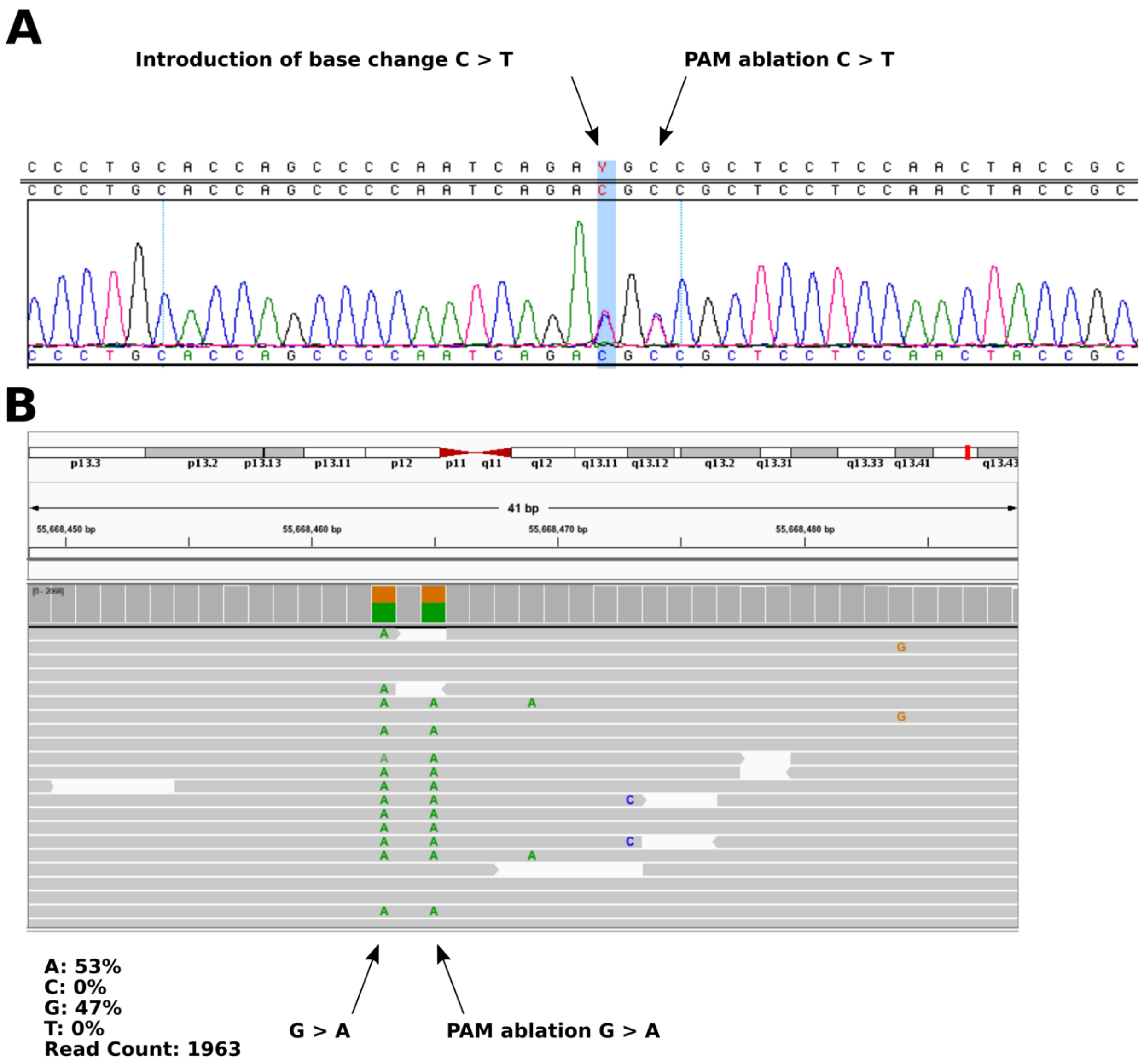

Online Figure V: Sequence of the *TNNI3*$^{R21C/+}$ iPSC line. A) Sanger sequencing trace 5'-3' across the intended mutation site, showing heterozygous introduction of C>T for the *TNNI3*$^{R21C/+}$ variant, with an additional PAM ablating silent mutation C>T to prevent further editing events. **B)** MiSeq reading 3'-5' showing the allelic balance of the variant across 1963 reads after an initial sub-clone of the identified variant, confirming the heterozygous *TNNI3*$^{R21C/+}$ variant.

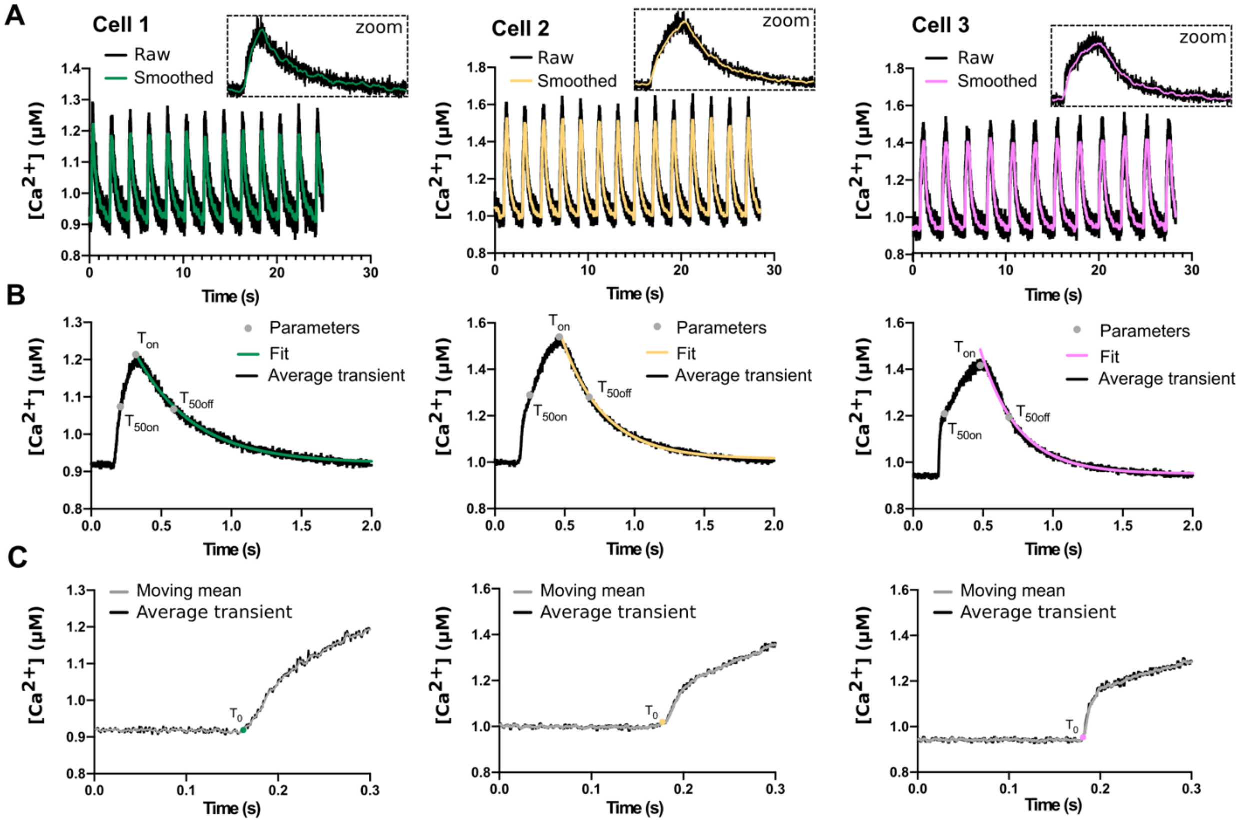

Online Figure VI: CalTrack processing of Fura 2 fluorescent traces acquired at 1000 FPS. Representative examples of three traces with distinctive steep (left), biphasic (right), and intermediate (middle) morphology. Raw fluorescence traces at 1000 FPS are smoothed down to 100 FPS for processing. Expanded view of single traces and their smoothed traces are shown in the inset. **B)** Average transients produced by reversion to 1000 FPS with parameter fitting. **C)** Expanded version illustrating robust detection of the initial point of calcium increase (T_0).

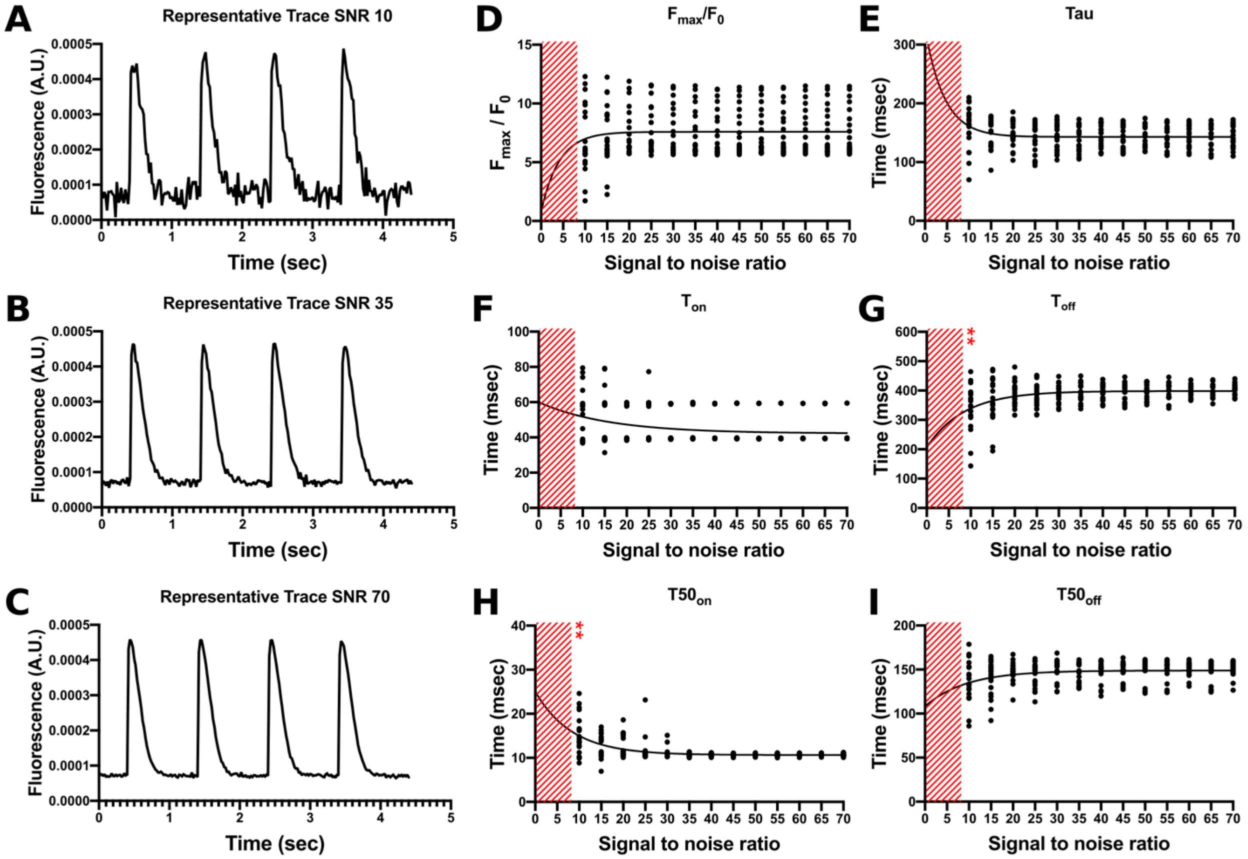

Online Figure VII: The effect of decreased trace signal to noise ratio on data measurement. A), B) and **C)** Representative simulation of signal to noise ratios at 10, 35 and 70 respectively on the same initial trace. **D)** Peak normalized RGECO fluorescence. **E)** Decay constant. **F)** Time to peak calcium. **G)** Time to complete calcium decay. Reduction to SNR of 10 decreased the time of decay. **H)** Time to 50% of maximum calcium. CalTrack measured $T50_{on}$ as increased at SNR 10. **I)** Time to 50% calcium decay. N = 20 for each signal to noise ratio and P values are reported as follows: p = 0.0043 for T_{off} and p = 0.0054 for T_{50on}, ** for $p \leq 0.01$ against SNR 70. Fitted lines represent nonlinear decay and increase fit. Red shaded section indicates traces that could not be analyzed with CalTrack. For F_{max}/F_0, T_{off} statistical analysis was performed by one-way ANOVA with post-hoc multiple comparisons (n=11) Sidak correction. Tau, T_{on}, $T50_{on}$ and $T50_{off}$ were tested with a paired non-parametric Friedman test with Dunn's multiple comparisons correction $p < 0.05$ was used as the significance cut-off.

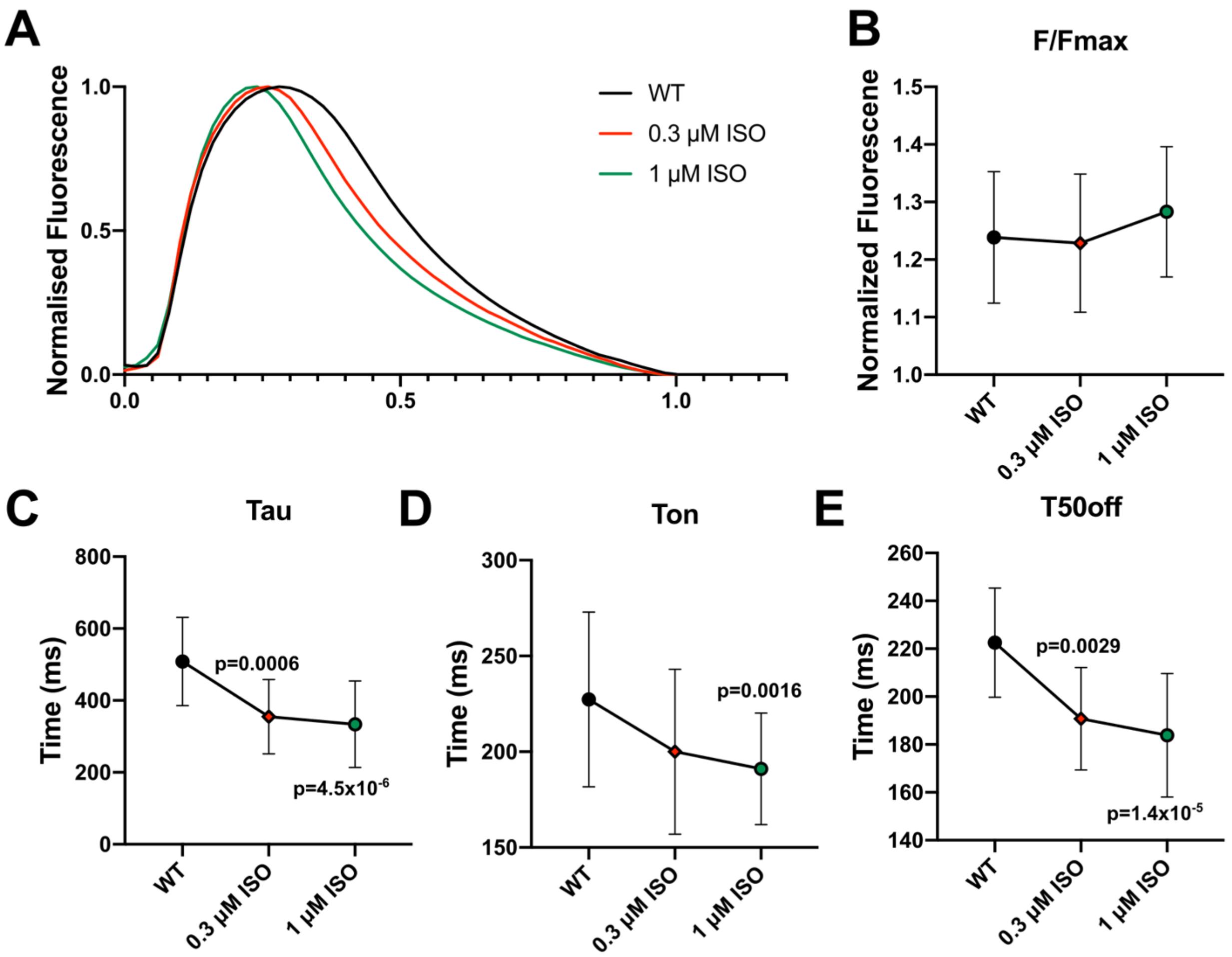

Online Figure VIII: Effect of isoproterenol treatment on WT iPSC cardiomyocytes. A) Normalized fluorescent calcium transients for untreated wild type (n = 34) at 0.3 µM (n = 38) and 1 µM isoproterenol (n = 71). **B)** Normalized peak fluorescence. **C**) Tau measurement illustrated more rapid calcium with increasing isoproterenol concentration. **D)** T_{on} decreased with isoproterenol treatment. **E)** $T50_{off}$ significantly decreased on application of isoproterenol. Statistical analysis of F/F_{max} and Tau was performed by one-way ANOVA with post-hoc multiple comparisons Sidak correction. T_{on} and $T50_{off}$ statistical analysis was tested by Kruskal-Wallis with a Dunn's multiple comparisons correction. $p < 0.05$ was used as the significance cut-off.

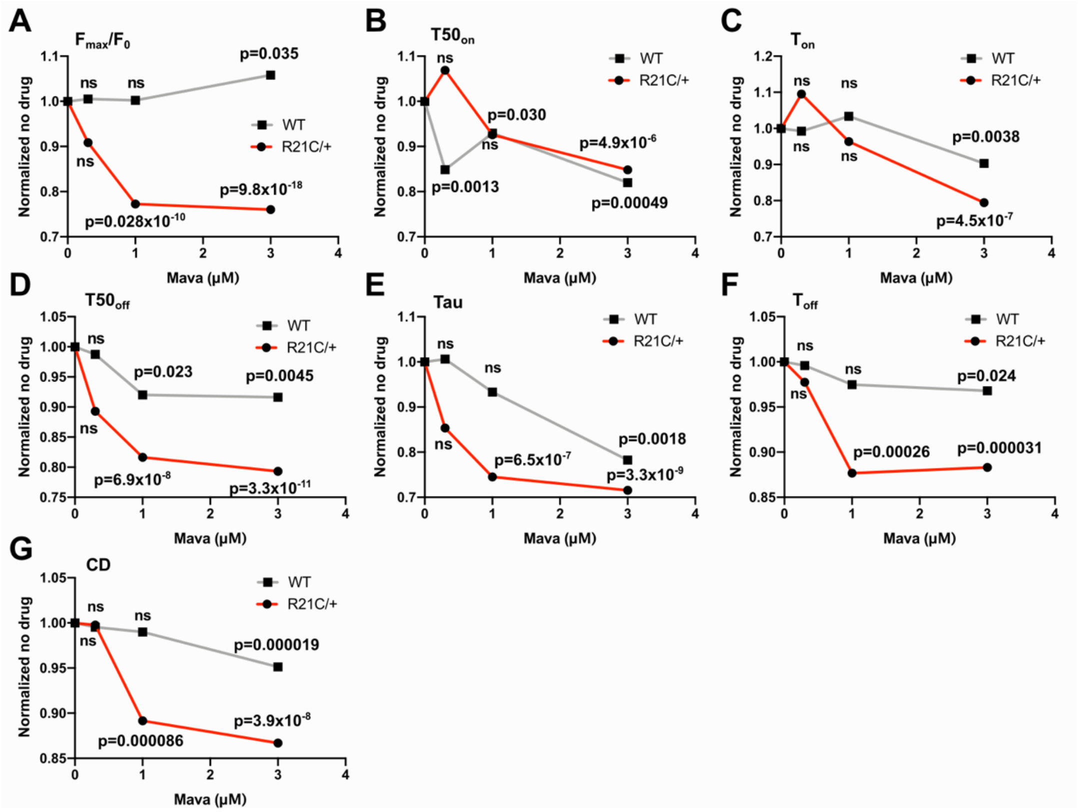

Online Figure IX: Effect of mavacamten treatment on the TNNI3 variant R21C and wild-type, each normalized to their respective control (0 µM point). Calcium parameters for WT (n = 82), WT + 0.3 µM Mava (n = 88), WT + 1 µM Mava (n = 90), WT + 3 µM Mava (n = 85), $TNNI3^{R21C/+}$ **(n = 60),** $TNNI3^{R21C/+}$ + 0.3 µM Mava (n = 40), $TNNI3^{R21C/+}$ + 1 µM Mava (n = 59) $TNNI3^{R21C/+}$ + 3 µM Mava (n = 92) . **A)** Normalized peak intensity. Mavacamten treatment sharply decreased peak normalized fluorescence for the mutant, indicating decreased calcium at peak in a dose dependent manner, but did not affect the WT except at extreme dosage whereby it increased. **B)** Time to 50% of calcium peak remained unchanged for both the mutant and WT up to 3 µM treatment, where $T50_{on}$ was achieved faster. **C)** Time to calcium peak remained unaffected except at maximum dose, when time to peak calcium was shorter. **D)** Time to 50% of calcium decay decreased for both in a dose dependent manner. **E)** The decay constant decreased at 3 µM for the WT, but decreased dose-dependently for the R21C variant. **F)** Time to complete calcium decay was unchanged in the WT but decreased in the mutant. **G)** Calcium transient duration decreased only at maximum dose in the WT but decreased in a dose-dependent manner in the mutant. Statistical analysis was performed by nonparametric Kruskal-Wallis with Dunn's correction for multiple comparisons (three comparisons). $p < 0.05$ was used as the significance cut-off.

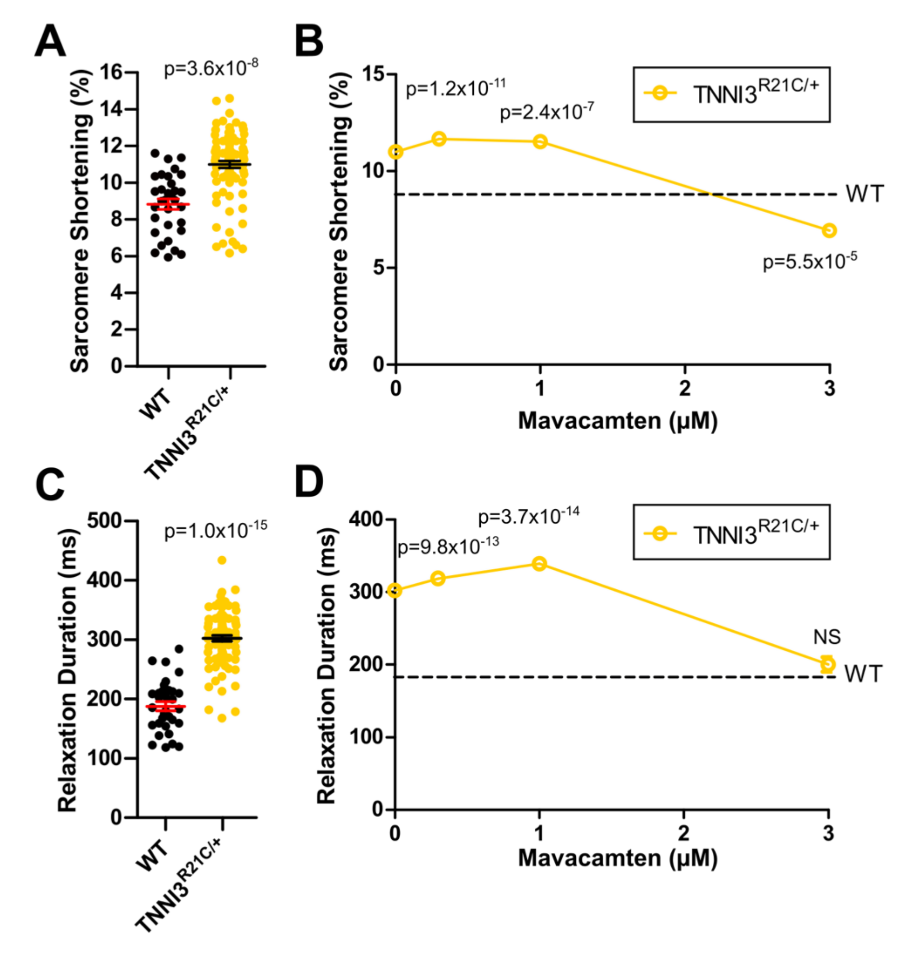

Online Figure X: Mavacamten corrects contractile changes caused by the TNNI3$^{R21C/+}$ variant in 1Hz paced iPSC-CMs, assessed by SarcTrack analysis. A) Sarcomeric shortening measured in WT (n = 33) and TnnI3$^{R21C/+}$ (n = 89) iPSC-CMs. **(B)** Mavacamten treatment of TNNI3$^{R21C/+}$ iPSC-CMs at 0.3 µM (n = 127),1 µM (n = 51) and 3 µM (n = 60). **C)** Relaxation duration increased in TNNI3$^{R21C/+}$ iPSC-CMs when compared to WT iPSC-CMs. **(D)** Mavacamten application at 0.3 µM (n = 127), 1 µM (n = 51), and 3 µM (n = 60) showed dose-dependent correction of relaxational abnormalities in the TNNI3$^{R21C/+}$ iPSC-CMs. Data presented as mean and standard deviation; statistical significances are denoted against WT. Normality was established using D'Agostino-Pearson. Students t-test were used in A) and C) where a significance cut off of $p < 0.05$ was applied. One-way ANOVA was employed to compare all Mavacamten treatments against WT on B) and D) with a post-hoc Sidak correction for multiple comparisons (three comparisons) with a significance cut-off of $p < 0.05$.

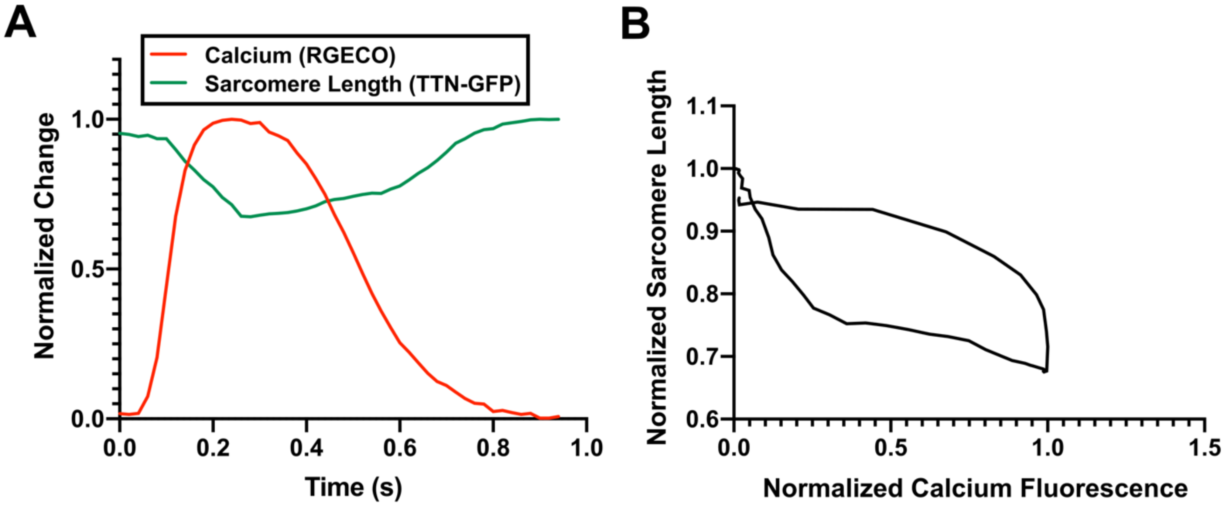

Online Figure XI: Simultaneous acquisition of sarcomeric motion and calcium fluorescence to illustrate the potential of CalTrack in conjunction with simultaneous sarcomere tracking. **A)** Average normalized calcium and sarcomere length. **B)** Sarcomere length – calcium loop.

ONLINE TABLE I. Comparisons of Currently Available Software for Calcium Transient Assessments

Method	Measurement parameters	Advantages	Limitations	Aimed at	Ref.
**SIMA**	Extraction of Ca^{2+} traces only	Motion correction, optional manual ROI editing	Parameter analysis dependent on external packages	Two-photon imaging of neuronal Ca^{2+}	*Kaifosh et.al PMID: 25295002*
**FluoroSNNAP**	Peak magnitude, inter-peak interval, baseline, time to 50% peak, decay time, cell-to-cell synchronisation	Can analyse single cells and temporal network patterns	Manual input for ROI selection required	Neuronal Ca^{2+} / Irregular Ca^{2+} characterization	*Patel et.al. PMID: 25629800*
**NeuroCa**	Peak magnitude, inter-peak interval, frequency, cell-to-cell propagation mapping	Baseline drift correction		Neuronal Ca^{2+} / Irregular Ca^{2+} Characterization	*Jang et.al. PMID: 26229973*
**PeakCaller**	Frequency, time to peak, time to baseline, synchronisation index, peak location, normalised peak height	Versatility of peak character, detrend facility	No inbuilt trace extraction from video microscopy, dependent on user for peak recognition	Neuronal Ca^{2+} / Irregular Ca^{2+} Characterization	*Artimovich et.al. PMID: 29037171*
**ClampFit**	Any temporal and magnitude parameter defined by the user, decay constant	Versatility of application	Intensive manual input, no automation	Electrophysiology traces Irregular trace Characterization	*Commercial source*
**Excel – based analysis**	Decay constant, absolute Ca^{2+} concentration, baseline, peak magnitude,		Intensive manual input, requirement of Visual Basic for Applications	Cardiac Ca^{2+} / regular Ca^{2+} characterization	*Greensmith et.al. PMID: 24125908*
**Cyt*o*Seer**	Frequency, peak magnitude, normalised area under the curve, duration of 25%, 50%, 75% and 90% of peak magnitude, time to peak and 50% decay	Baseline drift correction, separation of sub-cellular compartment transients, filtering of non-excitable cells and aberrant traces	Manual input for thresholding, proprietary	Cardiac Ca^{2+} / regular Ca^{2+} characterization	*Cerignoli et.al. and McKeithan et.al. PMID: 22926323*

Method	Measurement parameters	Advantages	Limitations	Aimed at	Ref.
**AxoGraph**	Any temporal and magnitude parameter defined by the user	Graphical interface	No automation, manual measurement of all parameters individually	Any trace in graph form	*Commercial source*
**Automated contractility/ Ca^{2+} MATLAB code**	Ca^{2+} trace extraction, unspecified Ca^{2+} temporal parameters, temporal correlation to contractile profile	Additional contractility assessment, user-friendly interface, open source, applicable to single and multiple cell constructs	Independent calcium analysis unavailable	Cardiac contractility & Ca^{2+} ; Standard Ca^{2+} characterization	*Huebsch et al. PMID 25333967*
**IonOptix IonWizard & MultiCell HTS**	Trace acquisition, measurement of baseline, including multiple parametric outputs.	Enables filtering and transient averaging, high throughput with MultiCell HTS	Proprietary, software only available with equipment, low throughput with IonWizard	Cardiac Ca^{2+} / regular Ca^{2+} characterization	*Commercial source*
**MATLAB ISA**	Peak fluorescent signal	Robust, tuneable cell segmentation	No Ca^{2+} character-ization	Non-excitable cells / Irregular Ca^{2+} patterns	*Wong et.al. PMID: 20952058*

Online Table II: Calculated parameters for multiple patterned iPSC-CMs and an iPSC derived Engineered Heart Tissue.

	F_{max}/F_0	CD (ms)	T_{on} (ms)	$T50_{on}$ (ms)	$T50_{off}$ (ms)	Tau (ms)	n	Analyzed by
**Patterned Cells (Werley et.al.)**	1.2 ± 0.015	664 ± 30	164 ± 4.8	41 ± 0.79	210 ± 18	319 ± 30	20	CalTrack Multicell
**EHT (Saleem et.al.)**	2.6	385	109	29	96	113	1	CalTrack SingleCell

Online Table III: Statical significances corresponding to Figure 8.

	$TNNI3^{R21C/+}$ + 0.3µM Mava vs $TNNI3^{R21C/+}$	$TNNI3^{R21C/+}$ + 1µM Mava vs $TNNI3^{R21C/+}$	$TNNI3^{R21C/+}$ + 3µM Mava vs $TNNI3^{R21C/+}$	WT vs $TNNI3^{R21C/+}$	WT vs $TNNI3^{R21C/+}$ + 0.3µM Mava	WT vs $TNNI3^{R21C/+}$ + 1µM Mava	WT vs $TNNI3^{R21C/+}$ + 3µM Mava	$TNNI3^{R21C/+}$ + Mava 0.3µM vs. $TNNI3^{R21C/+}$ + Mava 1µM	$TNNI3^{R21C/+}$ + Mava 0.3µM vs. $TNNI3^{R21C/+}$ + Mava 3µM	$TNNI3^{R21C/+}$ + Mava 1µM vs. $TNNI3^{R21C/+}$ + Mava 3µM
**Fmax/ F0**	**ns**	$2.8x 10^{-11}$ ****	$2.2x10^{-16}$ ****	**ns**	**ns**	$5.2x10^{-15}$ ****	$4.1x10^{-23}$ ****	$1.9x10^{-6}$ ***	$5.8x10^{-10}$ ****	**ns**
**Tau**	**ns**	0.030 *	$4.9x10^{-6}$ ****	0.0010 **	**ns**	$4.4x10^{-5}$ ****	$3.1x10^{-8}$ ****	0.0035 **	0.00067 ***	**ns**
**T50on**	**ns**	**ns**	$4.5x10^{-7}$ ****	$3.0x10^{-8}$ ****	**ns**	$8.2x10^{-8}$ ****	$2.2x10^{-17}$ ****	0.0023 **	$4.1x10^{-7}$ ****	**ns**
**Ton**	**ns**	$6.9x10^{-8}$ ****	$3.3x10^{-11}$ ****	$2.2x10^{-16}$ ****	0.00020 ***	$2.9x10^{-11}$ ****	$5.5x10^{-32}$ ****	0.025 *	$5.3x10^{-9}$ ****	0.00094 ***
**T50off**	**ns**	$6.5x10^{-7}$ ****	$3.3x10^{-9}$ ****	0.044 *	**ns**	$2.7x10^{-8}$ ****	$6.3x10^{-14}$ ****	0.010 *	0.0010 **	**ns**
**Toff**	**ns**	0.00026 ***	$3.1x10^{-5}$ ****	$1.7x10^{-5}$ ****	0.00045 ***	**ns**	Ns	0.0060 **	0.0023 **	**ns**
**CD**	**ns**	$8.6x10^{-5}$ ****	$3.9x10^{-8}$ ****	0.035 *	**ns**	$6.2x10^{-5}$ ****	$5.6x10^{-10}$ ****	0.00010 ***	$1.7x10^{-7}$ ****	**ns**

Nonparametric Kruskal-Wallis with Dunn's correction for multiple comparisons (three comparison against genotype). Nonparametric Kruskal-Wallis with Dunn's correction for multiple comparisons (three comparison against WT). Nonparametric Kruskal-Wallis with Dunn's correction for multiple comparisons (three comparison among all columns). **Nonparametric Mann-Whitney test.**

ONLINE ONLY VIDEOS:

Supplemental Movie I: Single iPSC-CM calcium transient measured by the RGECO calcium sensor. Imaged using a 100X oil immersion lens; electrically paced at 1Hz. Fluorescence calcium transients acquired at 40 FPS.

Supplemental Movie II: Multiple guinea pig cardiomyocytes with fluorescent calcium transients using the RGECO calcium sensor. Imaged using a 20X objective, and electrically paced at 0.5 Hz. Fluorescent calcium transients are acquired at 25 FPS.

Supplemental Movie III: Simultaneous imaging of iPSC-CM for calcium using RGECO (left) and sarcomere shortening using TTN-GFP (right). Images using a 100X objective, and electrically paced at 1 Hz.
