# Supplementary material for: CalTrack: High-Throughput Automated Calcium Transient Analysis in Cardiomyocytes
Source: Circ Res. 2021 May 21;129(2):326–41. doi: 10.1161/CIRCRESAHA.121.318868 (PMC8260473; doi:10.1161/CIRCRESAHA.121.318868)
Supplement: Supplementary file 7 [file res-129-326-s007.pdf]

SUPPLEMENTAL MATERIAL

CalTrack User Guide

1) CalTrack can be downloaded from https://github.com/ToepferLab/CalTrack. This package includes two algorithms to assess calcium transients in different cell types and formats called "Single Cell" or "Multi Cell" (See main text Figure 1).
2) CalTrack relies on the use of the Bio-Formats toolbox for MatLab to read the microscopy data. The user needs to download the Bio-Formats MatLab toolbox from this URL https://www.openmicroscopy.org/bio-formats/downloads/ (click on the "MATLAB toolbox" to obtain a folder called "bfmatlab"). The obtained bfmatlab folder has to be selected by the user when CalTrack asks to do so. Additional information on how Bio-Formats can be called from MatLab can be found here https://docs.openmicroscopy.org/bio-formats/6.5.0/users/matlab/index.html.
3) The user must have the MatLab Curve Fitting and Image Processing Toolboxes installed.
4) In order for CalTrack to analyze the videos, the videos' names have to be less than 31 characters and include no special characters.

CalTrack consists of algorithms that allow the analysis of intracellular calcium in different cell types. The algorithms are divided in two folders called "Single Cell" and "Multi Cell". However, to make the use of CalTrack as user friendly as possible, the code is structured so that the user needs to run only one script called "main.m" and follow the instructions prompted through dialog and question boxes.

This user guide provides information on how to run CalTrack using the main.m script and also on the underlying algorithms.

Main.m

The main.m script is the only script that the user needs to run to perform the analysis.

First, main.m determines whether the "Single Cell" or "Multi Cell" algorithm is appropriate for the analysis by prompting the user to select if single or multiple cells are to be analyzed through a question dialog box. Then, an input dialog box prompts the user to input the parameters of

acquisition that need to be defined for each data set. These are (i) the acquisition frequency of the videos, (ii) the pacing frequency of the cells to be analyzed, (iii) the number of frames to be discarded at the beginning of each movie, (iv) the last frame to be analyzed, (v) the threshold for defining extra beats or non-adherence to pacing, as a percentage tolerance threshold with relation to the frequency of electrical pacing, (vi) whether the calcium parameters should be also computed on the individual beats of each trace, (vii) whether calcium traces need to be extracted from the videos, (viii) whether the user wants to provide the frame number corresponding to electrical pacing and use that information to segment transients, and (ix) whether the fluorescent traces should be converted to calcium values (a calibration curve is needed for this; further details are provided below). Question (vii) is asked because extracting calcium traces from the videos is the most time-consuming step of the algorithm; should the user already have the traces in the right format (a MatLab structure called "Calcium_Traces.mat" for the single cell algorithm, or an excel file called "CalciumData_MultiCell.xlsx" for the multi cell algorithm), then this step can be skipped by replying "n" to this question. We also provide a separate MatLab function "xlsx_to_mat.m" that allows the user to convert excel files into "Calcium_Traces.mat" MatLab structures.

CalTrack also allows the user to save previously entered analysis parameters so that they can be used for subsequent analyses without the need to re-enter parameters.

After asking for the parameters of acquisition, CalTrack will then prompt the user to select the calcium traces, whereby analysis will proceed.

Based on the inputs provided by the user, main.m will run different scripts sequentially: "CalTrack.m", "FR_to_calcium.m", "DataAnalysis.m", "PlotParameters.m" and "NonRegularBeatsAnalysis.m". Description of each of these scripts is provided below. During this process, a few more questions will be prompted to the user to ensure that the analysis is successful.

Single Cell

Scripts

These scripts are run sequentially. Initially CalTrack.m is run to extract calcium traces from the images. "FR_to_calcium.m" is run if the user has selected the option to convert fluorescent traces in calcium values. DataAnalysis.m identifies and averages calcium transients to a single average

calcium transient. This average transient is then used to perform fitting of the decay, allowing the measurement of tau (τ) (the other fitting parameters *a* and *c*, and the coefficient of determination (R-squared) as goodness-of-fit statistics are also reported), and to compute F_{max}/F_0, baseline value, and multiple temporal measurements of the calcium transient (CD, CD90, CD50, CD10, T_{on}, T_{off}, $T10_{on}$, $T50_{on}$, $T90_{on}$, $T10_{off}$, $T50_{off}$, $T90_{off}$, BR, t0, tend, BBtime_mean, Nperiod). The signal to noise ratio is also reported. The description of these parameters can be found below.

1. CalTrack.m

This script is run if the user answers "y" to the question "would you like to extract traces from videos?" in the input dialog box described above. It involves the following steps:

- The user is asked to select the directory that contains the Bio-Formats folder (bfmatlab), so it can be added to the MatLab path.
- The user is then asked if files need to be converted to .tif by a yes/no dialogue entry. This includes proprietary microscopy formats such as .vsi and .czi files. If the user opts to convert files, they are prompted to select the directory where the microscopy files are located, followed by the current format of the files.
- If the user opts not to convert files to .tif, they are asked to select the directory where the .tif image stacks are, since the algorithm works best with .tif files for optimal retention of information. The algorithm subsequently extracts (from .tif files) the corresponding fluorescent trace from each image stack and saves all traces in a MatLab structure named "Calcium_Traces" in the folder "calcium_analysis". This directory is created automatically in the folder where the original movies or .tif stacks are located to enable organized archiving.

In order for the algorithm to extract the fluorescent traces from image stacks, image processing is carried out and morphological operators are applied to segment the cells and exclude the background. Examples of the masks used to segment the cell are reported in Figure 1.

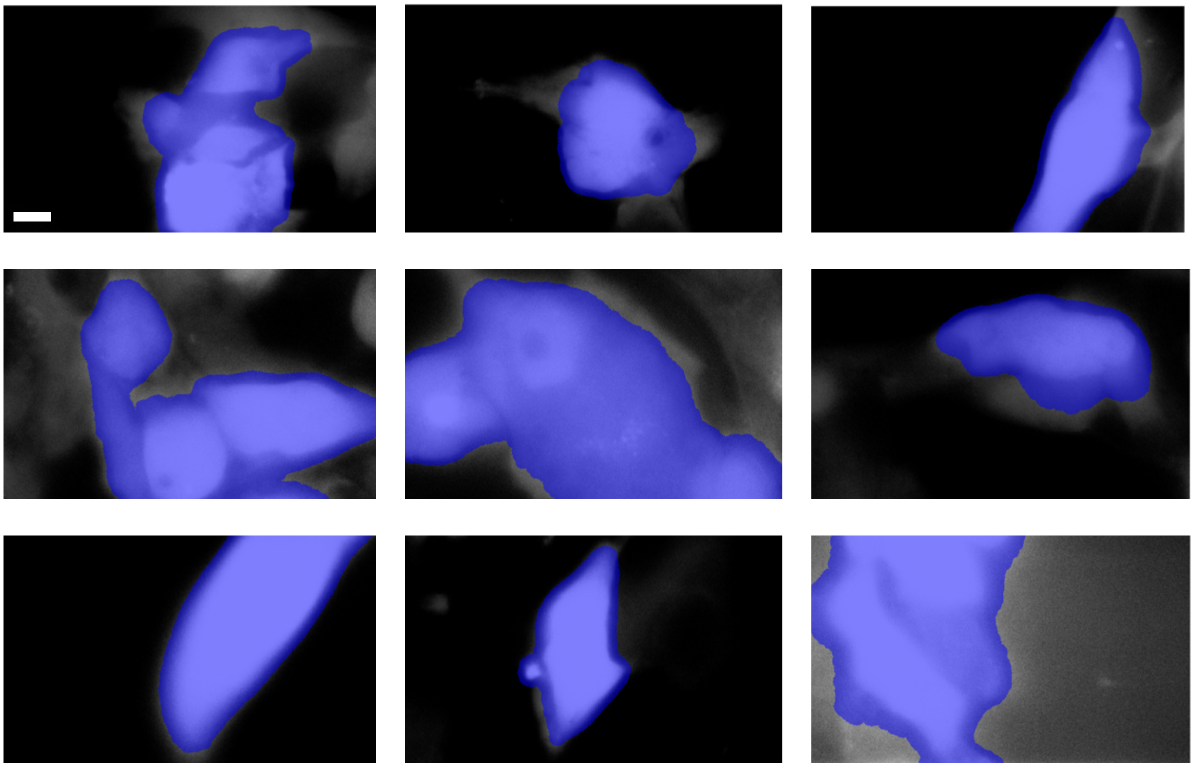

Figure 1. Examples of segmented cells. The white bar is 10 μm

2. DataAnalysis.m

DataAnalysis.m generates the averaged fluorescent transient, based on the whole fluorescent trace generated in CalTrack.m. Parameters are subsequently extracted from the averaged calcium transient. Note that τ is computed through fitting of the decay part of the trace, and the remaining calcium transient parameters are calculated by linear interpolation of the average transient. The average transient of each cell, and parameters, are saved in excel files in the folder "calcium_analysis". The algorithm is also capable of computing the parameters on each individual transient of a fluorescent trace. If the user opts to do so, the results are saved in a separate excel file.

N.B. Not every calcium trace is automatically analyzed. Traces excluded from automatic analysis are those too noisy for reliable averaging, those that present extra beats/non-adherence to pacing (outside the percentage tolerance limit defined by the user), or those that present early afterdepolarization/delayed afterdepolarization-like events.

DataAnalysis.m generates plots to aid the user in their analysis. The first plot shows the fluorescent traces that will be analyzed in red (i.e. based on the number of frames provided by the user, these are the portions of the whole trace that the user has specified for analysis) compared to the original ones, drawn in blue (Figure 2). If the user decided to discard 1 frame at the beginning of the movie and then to analyze 500 frames in total, then the algorithm will analyze the calcium trace from frame number 1 to frame number 500. This option has been implemented to enable analysis of cropped traces, for example where long traces are acquired at multiple pacing frequencies.

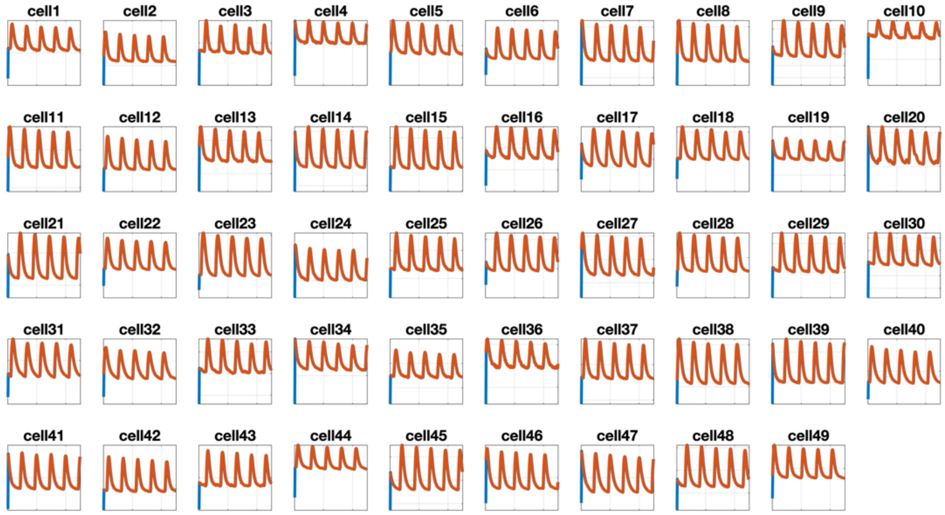

Figure 2. First plot in the algorithm. Original traces in blue and traces that will be analyzed in red. In this example, the first frame has been removed from each trace. Each subplot represents a different cell. The algorithm does not provide x or y labels in this figure as the important information at this stage is the comparison between waveforms.

The user can decide, based on Figure 2, to discard any of the calcium traces. Then, the user is prompted to decide if the traces to be subsequently analyzed require photobleach correction. Briefly, traces exhibiting linear or exponential increase, or decrease, across the entire trace can be de-trended to return the trace to an even baseline.
Next, the user is presented the corrected traces if photobleach correction is selected and asked to decide whether any traces should be discarded.
Finally, the fluorescent trace of each cell is subsequently divided in single beats which are then averaged into a single composite transient. This is presented to the user with a prompt to discard any cells if they choose so, for quality control purposes.
Single composite transients are subsequently used to compute the calcium transient parameters.

Beat segmentation is performed through the following steps:

y is the total fluorescence of one cell (featuring multiple transients). In order to separate each beat, high-fidelity identification of the starting point of each transient is necessary. This is done by computing the "*diff*" of the signal (difference between consecutive points) since the rise of each transient is characterized by a significant increase compared with the previous point, manifesting as a prominent peak (Figure 3).

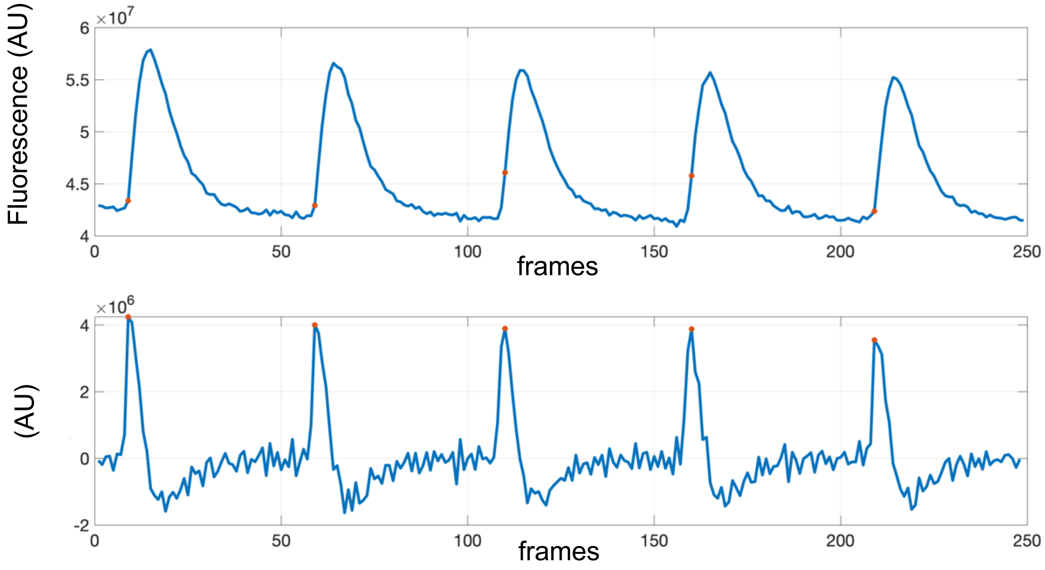

Figure 3. First panel: signal y. Second panel: diff signal. Red dots represent the peaks of the diff signal, and, as shown in the first panel, they help identifying the beginning of each transient.

The algorithm then searches for the peaks in the signal y_diff with the MatLab function "findpeaks". This detects all peaks in the trace and is filtered to only consider those that have a prominence of at least 50% of the peak with maximum prominence.

The algorithm then checks that the signal is not too noisy to be automatically analyzed, since this will preclude prominent peak detection with the function "findpeaks". In this scenario, the algorithm "skips" noisy cells and saves them in a structure named "skipped" so that they are flagged to the user. Noisy signals are identified by computing the peaks of the y signal and then comparing the number of these peaks with the number of peaks in diff_y. If the number of peaks in diff_y signal is greater than the number of peaks of the y signal (+2), then the signal is too noisy for accurate analysis and excluded. Comparing against the number of peaks in the original signal is more robust and informs the user on the number of beats that are in the trace, however, this number may also include incomplete beats at the beginning and end of the trace. To overcome this the algorithm employs *(length(peaks_diff) - length(peaks_y)) > 2* in order to account for potential incomplete transients detected at the beginning and at the end of the signal.

The algorithm subsequently checks that the peaks of the signal y correspond to the pacing frequency. If two peaks are closer than the pacing period (1/pacing frequency), the trace is classified as having extra beats. This associates with the user-defined threshold for extra beats. The threshold is given as % of the pacing frequency. For example, a 10% threshold for 1 Hz pacing means that only beats with a shorter periodicity than 0.9s are considered extra beats. Traces with extra beats are saved in a separate MatLab structure, named "extra_beat", along with information on the name of the cell, the average periodicity of peaks and the number of beat-to-beat periods used to compute that average. Note that this step uses the peaks of the signal y, while the next step to identify the single beats uses the peaks of the signal diff_y which identifies the rising phase of the transient. If an extra beat or beats are detected, the trace is removed from further downstream analysis as dividing the trace into single beats will not be accurate.

For signals that successfully pass through automatic analysis, each beat is identified from the y signal as going from one peak-offset to the following peak-offset (peak of the diff_y signal). The offset is set to correspond to 20% of the pacing period and represents the number of points that are included in a single transient before its rising phase (identified using the peaks of the *diff* signal). In this way a trace that goes from baseline to baseline is generated (Figure 4).

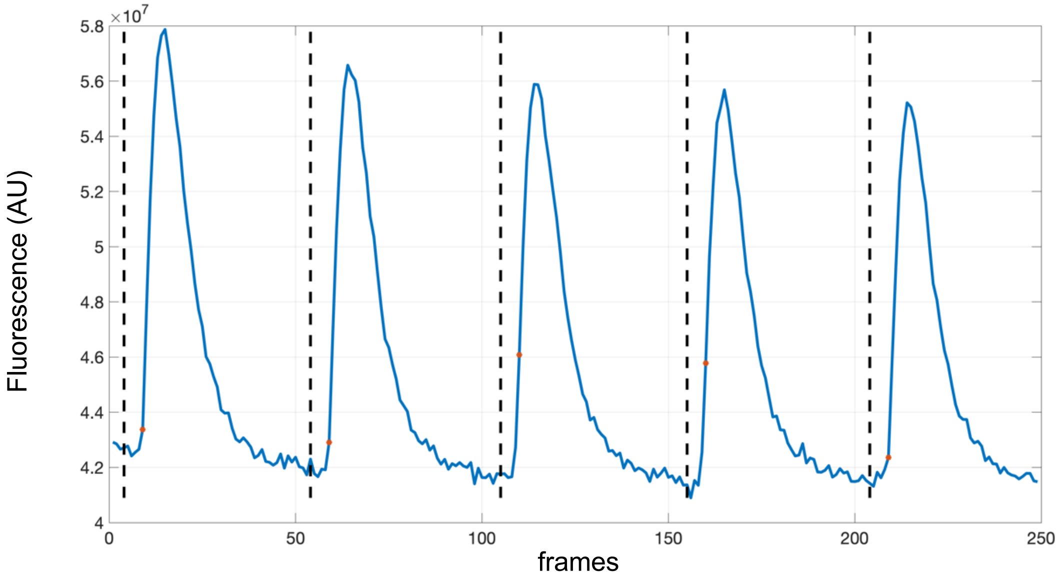

Figure 4. Identification of each beat. The x coordinate of dashed lines is computed from the red dots (peaks of the diff signal) by subtracting the offset.

Note:

- If there are no offset points before the first peak, the first transient will start from the beginning of the signal.
- If the first transient doesn't start at the baseline, it is not detected by the "findpeaks" function (or if there is no change in the slope of the rise phase of the transient), as shown in Figure 5.

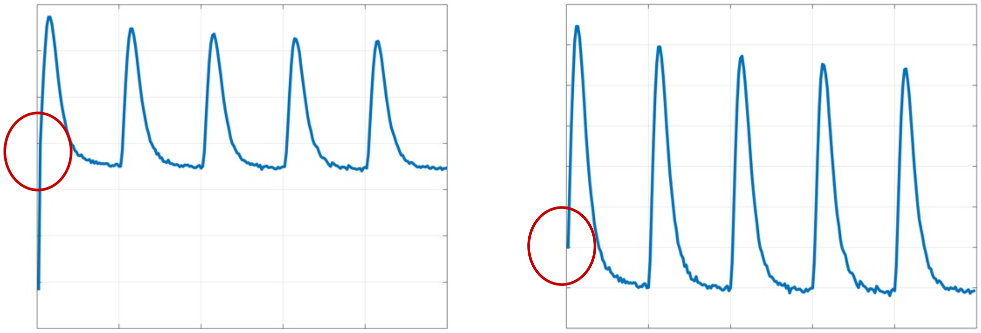

Figure 5. Examples of first transients not detected.

- The last transient, which goes from the last peak-offset to the end of the trace, must be considered on a special-case basis as two problems may be encountered:
  1. The last transient may not return back to baseline (i.e. it's incomplete, Figure 6). Therefore, it is removed. The last point of the last transient needs to be equal or smaller than the first point of that last transient.

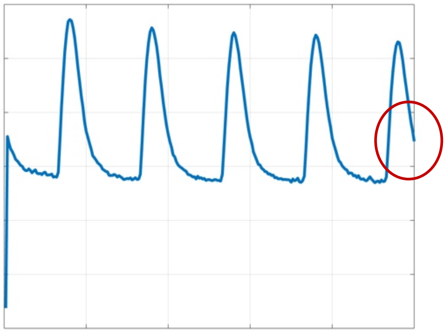

Figure 6. Last transient is removed because it is incomplete.

2. The last transient is complete, but the final part has to be removed (Figure 7). In this case, the algorithm will perceive the last transient to be as shown inside the black box. The final part of that transient, circled in red, must therefore be removed.

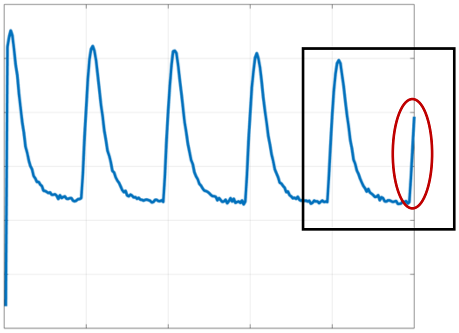

Figure 7. Last transient (black box) is kept but the final part (red circle) is removed.

To do this, the algorithm works specifically on the last transient. The first element is added to the last one and then checked for the presence of more than 1 peak (adding the first element is necessary, otherwise the second peak is not detected since it is the last element of the array; this is because the diff_y signal is the difference between consecutive points). If there is only one peak, the algorithm perceives this to be scenario 1. If there is more than 1 peak, the signal is cropped from the beginning to the second peak-offset.

Once the beats have been identified and separated, the beat rate (BR) is computed as the number of beats divided by the time vector to obtain the number of beats per second. This should equal the pacing frequency if the cells are not deviating by spontaneously depolarizing at a faster frequency, or not responding to electrical pacing.
The time vector (in seconds) is now a vector ranging from 1 to N, where N is the sum of the lengths of all beats, multiplied by the frame time (1/acquisition frequency). The number of beats detected, divided by this time vector, gives the number of peaks per second (BR).

If any error occurs in trying to isolate single beats, the whole curve is saved in a separate excel file "errors" for the user to visualize.

In summary, there are three types of traces that are not automatically analyzed:

- Transients with high noise where peaks cannot be robustly identified.
- Traces where there is non-adherence to pacing or to a specific beat rate (as input by the user).
- Non-specific error (rare and can be caused by a number of technical inconsistencies with the initial acquired image stack).

The following MatLab structures are created and used for subsequent analyses and output export:
single_traces: data that go through automatic analysis
extra_beat: extra beat traces
skipped: traces skipped due to noise
errors: traces skipped due to errors
errors_parameters: traces skipped due to errors during parameter computation (expanded below).

If photobleach correction was selected, it is performed after beat segmentation (using the function PhotoBleachCorrection.m). The corrected traces are then used to perform beat segmentation again (this is because trace correction requires segmented beats to determine the baseline trend). Corrected traces are plotted against their respective original traces, with the option to discard any traces not adequately corrected.

Once initial traces are segmented, a second plot (Figure 8) is displayed, which shows all segmented traces for each individual image stack so that the user can visually check that the individual beats have been appropriately segmented. If not, the user can decide to discard any cell.

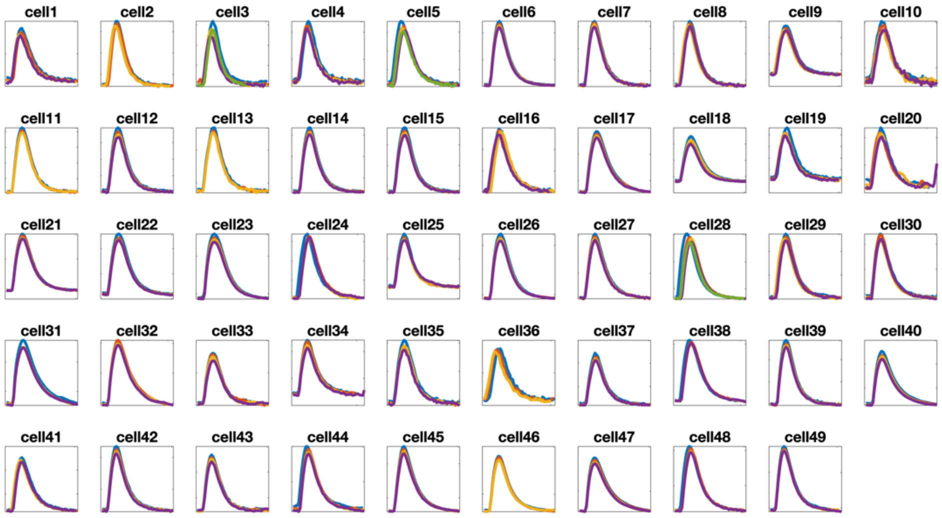

Figure 8. Second plot of the algorithm. For each cell, the segmented transients are plotted to allow the user to verify that the segmentation process was successful. Each subplot represents segmented traces from a different image stack.

The structure "single_traces" is generated and contains the single segmented transients for each cell. Each set of transients from a single image stack is averaged to the transient of shortest length (Figure 9) and saved in the structure named "single_traces".

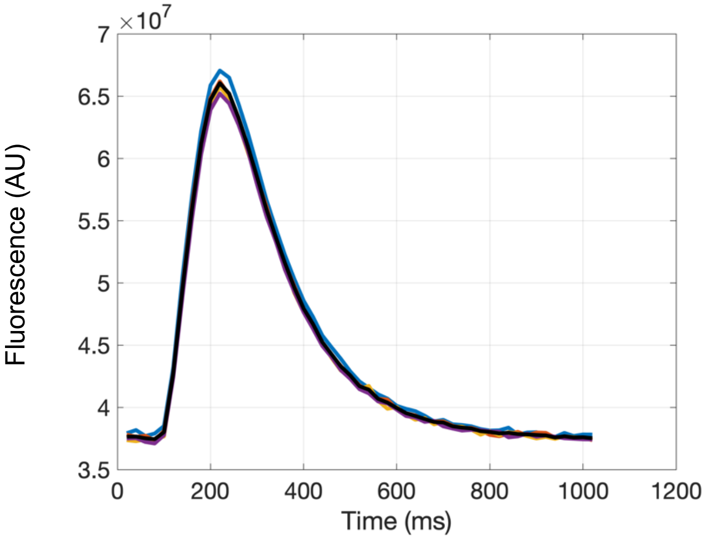

Figure 9. For each image stack, an average single-beat transient is computed (black) and used to compute the calcium parameters and fitting.

In the scenario where the user opts to compute the parameters on each individual beat and then average the computed parameters, these are saved in the excel file "Calcium_measurements_AvgParam".

On completion, parameters and fitting are additionally computed on the average transient (generated as described above). From this average transient, τ is computed by fitting the decay part of the signal using the function $y = ae^{bx} + ce^{dx}$, where $d = 0$ and $b = 1/(-\tau)$.

For each average trace the following parameters are computed:

Baseline (AU): also called F_0, mean value of the last points of the average transient that correspond to a temporal window of 20% of the pacing period. Given as an arbitrary unit of total fluorescence unless the user selected to convert fluorescence into calcium values by providing a calibration curve, in this case this is μM.

F_{max}/F_0: maximum fluorescence value (F_{max}) divided by baseline value (Baseline or F_0).

For the remaining parameters, a different baseline value is considered. This is computed from the original baseline described above, as baseline + 0.03(F_{max}-baseline).*

CD (ms): time of fluorescence (signifying calcium) above baseline, i.e. duration of the calcium transient.

CD90 (ms): time calcium is above 90% of the signal (defined as baseline + 0.1*magnitude).

CD50 (ms): time calcium is above 50% of the signal (defined as baseline + 0.5*magnitude).

CD10 (ms): time calcium is above 10% of the signal (defined as baseline + 0.9*magnitude).

T_{on} (ms): time at which calcium is maximal, computed from t_0.

T_{off} (ms): time from peak to tend.

$T10_{on}$ (ms): from t_0, time to 10% contraction (defined as baseline + 0.1*magnitude).

$T50_{on}$ (ms): from t_0, time to 50% contraction (defined as baseline + 0.5*magnitude).

$T90_{on}$ (ms): from t_0, time to 90% contraction (defined as baseline + 0.9*magnitude).

$T10_{off}$ (ms): from peak, time to 10% relaxation (defined as baseline + 0.9*magnitude).

$T50_{off}$ (ms): from peak, time to 50% relaxation (defined as baseline + 0.5*magnitude).

$T90_{off}$ (ms): from peak, time to 90% relaxation (defined as baseline + 0.1*magnitude).

Tau (ms): generated from fitting signal y to $y = ae^{bx} + c$. tau = 1/-b.

fit_a: parameter a of the fit.

fit_c: parameter c of the fit.

fit_rsquare: goodness-of-fit, coefficient of determination (R-squared).

BR (Hz): beat rate.

t_0: obtained as the intersection between the signal (before peak) and baseline.

t_{end}: obtained as the intersection between the signal (after peak) and baseline.

BBtime_mean (ms): average beat-to-beat time.

Nperiod: number of periods used to compute that average.

Signal to noise ratio: average transient value divided by the standard deviation of baseline

Two additional parameters (BBtime_mean_eb and Nperiod_eb) are reported only for the calcium traces for which extra beats have been identified.

The calcium amplitude (CaAmplitude) is also computed as the difference between peak and original baseline and reported as output parameter (this is especially useful if fluorescent ratios are converted in calcium concentrations).

If an error occurs in computing the parameters, the specific problematic curve is saved in a separate excel file "error_parameters" for the user to visualize if needed. The outputs (traces and parameters) are stored in excel files. Traces are exported so that they all have the same length, meaning that for the shortest ones their final value is replicated to match the length of the longest trace.

If the user opted computation of calcium parameters on each individual beat, DataAnalysis.m performs this task before computing the average transient and parameters as described above. Specifically, after beat segmentation each individual beat is used to compute the calcium parameters (these are then averaged and reported in "Calcium_Measurements_AvgParam.xlsx"). For each cell the parameters' standard deviation between beats is also collected in a matrix and output in the second sheet of the excel file "Calcium_Measurements_AvgParam". Here, the first column is the number of beats used to compute the standard deviation. This provides the user a quantification of how similar individual beats were before averaging them (i.e., the transient's regularity). Furthermore, each individual beat is inspected for early afterdepolarization (EAD) and delayed afterdepolarization (DAD)-like events. If these are detected, the corresponding beats are excluded from transient averaging and saved in a separate excel file "Calcium_Traces_NonRegularBeats.xlsx" with information on the number of cells affected, number of regular transients and the number of EADs and DADs per cell. We define the presence of EAD-like events as the detection of peaks in the decay phase of the transient, with prominence larger than 0.2*decay amplitude. We define the presence of DAD-like events as the detection of peaks in the baseline (following the decay phase) of a transient with prominence larger than 0.03*baseline mean.

If the file "Calcium_Traces_NonRegularBeats.xlsx" is generated, then the cells included in the file can be further analyzed by the function "NonRegularBeatsAnalysis.m" as explained below. The user is asked whether they want to perform this analysis through a question box.

The calcium parameters computed on each individual beat and the corresponding beat trace are reported in the excel files "Calcium_measurements_IndividualBeat.xlsx" and

“Calcium_Traces_IndividualBeat.xlsx”, where each excel sheet represents a different cell and each row/column a different beat.

3. PlotParameters.m

This script is only run if the user opts to plot the measured results on prompt by the dialog box “Would you like to plot the results?”. The user can visualize whether the parameters and fitting are computed correctly by automatically plotting all parameters on their respective average transient. The script loads the excel files output from DataAnalysis.m and generates three plots illustrating the average transient of each cell and its corresponding parameters. It also displays on the command window the number of cells that have gone through automated analysis, and the number of cells discarded for one of the following reasons: transients with high noise, experiments where there was non-adherence to pacing or a specific beat rate, scenarios where non-specific error occurred, and scenarios where errors occurred while computing the parameters.

Importantly, when extra beats are detected in an image stack by CalTrack, the output excel file records a row of zeros for each parameter of that image stack but the final two (BBtime_mean and Nperiod). PlotParameters removes these rows so that the plots generated show only the parameters of the cells that were successfully analyzed automatedly.

For each of the cases mentioned above (too much noise in the signal, extra beat or beats detected, and occurring errors), if any, plots are generated to display the curves.

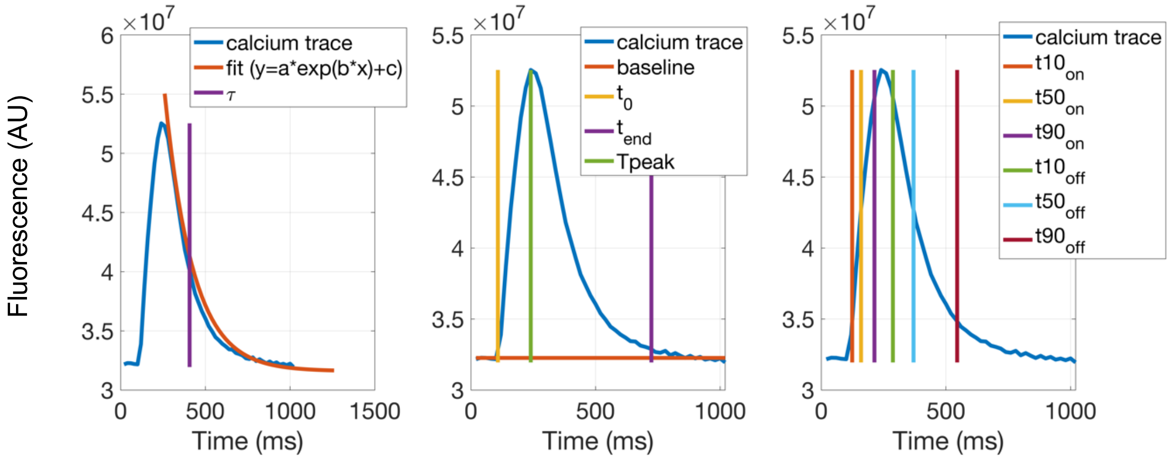

Figure 10. In PlotParameters.m, for each image stack that goes through the automated analysis, the average transient with corresponding parameters is plotted.

Functions

Below we outline the core functions that support the scripts CalTrack and DataAnalysis.

convert_to_tif

This function is used by CalTrack and converts video files into .tif. The file format is subject to the user's input.

single_stack_loader

This function is used by convert_to_tif and loads a dual color stack using bformats.

BeatSegmentation.m

This function is used by DataAnalysis and given a multi-beat trace, segments the individual beats.

PhotoBleachCorrection.m

This function is used by DataAnalysis and given a segmented multi-beat trace, preforms the photobleach correction.

parameters.m

This function is used by DataAnalysis and given a single-beat trace, computes the parameters.

intersection.m

This function is used by parameters.m and computes intersections of curves.

Additional analysis features for specific scenarios:

NonRegularBeatsAnalysis.m

A prerequisite for this analysis is that the signal to noise ratio of the traces is high enough to work with non-averaged transients.

Briefly, CalTrack separates regular from non-regular behaviors and automatedly analyzes regular calcium transients. For the regular transients, it averages them and uses the average transient to compute the calcium parameters as described above. In addition to this, it can also compute the same parameters on each individual transient and from these it can provide the standard deviation between transients.

The traces identified as having non-regular (non-adherence to pacing or presence of spurious beats) behavior are saved in additional excel files (extra_beats and NonRegularBeats). These files can be read by the MatLab script NonRegularBeatsAnalysis.m which analyzes these traces by excluding the non-regular beats and providing the calcium parameters of the regular ones.

Non-regular behavior may include:

- Extra beats (non-adherence to pacing). These traces have peaks with prominence of at least 50% the prominence of the highest peak and lie closer to their preceding peak than expected based on the pacing frequency. These are likely to be “real beats” that are just not following the electrical pacing. Any spurious peak that has a prominence less than 50% of max prominence is not detected as extra beat.
- Beats with spurious peaks (EAD/DAD-like events). To detect these, we evaluate the decay of each beat (EAD) and the baseline trace of each beat (DAD) in the same way as for extra beats.

The separate analysis of irregular behaviors is able to:

- for non-adherence to pacing, plot the multi-beat traces, and provide the number of beats excluded because their beat-to-beat interval is different than expected. Also, after removing beats for being too close, the algorithm checks that the remaining beats do not have any irregular peaks (EADs/DADs). If they have irregular peaks, these transients are excluded as well. Finally, the remaining regular transients are used to compute the parameters.
- For the EAD/DAD-like events, plot the original multi-beat trace, and the segmented beat that showed irregular behavior, and provide the cell and beat number for the irregular behavior. It then excludes the irregular beat(s) and compute parameters on the regular ones (new excel files).

Non regular transients are excluded from the averaging process and parameter computation.
The standard deviation between beats is computed by considering the beats that did not show irregular peaks, as a quality control measurement of how similar the beats we averaged to get the mean transients were.

FR_to_calcium.m

This MatLab function is provided to convert fluorescence ratios (FR) to calcium concentrations for the calcium indicator Fura 2. For this, a calibration curve is needed, and is assumed to be

$$[Ca] = -a + b * FR^c$$

The user can either provide the values for a, b, and c (all three assumed to be positive) or can provide a series of [Ca] and corresponding FR points. For the latter case, CalTrack will fit the above equation to the provided points to estimate a, b, and c.

To convert Fura 2 data into calcium concentrations the user can select this option in the initial dialog box and provide the calibration curve in either an excel file “CalibrationCurve.xlsx” where the three parameters mentioned above are specified, or in a “CalibrationPoints.xlsx” file where the [Ca] and corresponding FR points are specified. Please organize these files as shown in the examples in the CalTrack folder provided with this guide.

xlsx_to_mat.m

In the CalTrack folder we provide a MatLab function xlsx_to_mat that can be used to convert excel files with multi-beat calcium traces (obtained with RGECO or Fura 2) into MatLab structures that be directly used within CalTrack.

Please organize you excel file as the one provided in this folder as an example (example_FuraData.xlsx). Please do not insert spaces in the name of the excel sheets (e.g. use "WT_data" and not "WT data"). All the excel sheets will be read by the MatLab function, so sheets with the average traces should not be included here.

The function will ask the user to select the excel file to be converted and then will create a series of folders where the new MatLab structures (one for each excel sheet, which are assumed to be a full data set each) will be saved. Then CalTrack can be pointed to each of those folders and will save the corresponding results there.

To run the function, simply type the following in the MatLab command window:
xlsx_to_mat()

Example:
Create a folder "data" and copy the excel files for the calcium data and the calibration file (if you have Fura 2 data) in it. Open MatLab, select the CalTrack folder as the current MatLab folder, and type xlsx_to_mat() in the command window. Copy the excel file for the calibration curve (if you need it) in each of the newly generated folders (which contain the Calcium_Traces.mat structures needed for CalTrack). In this way your original excel file could also contain cells that have different calibration curves. Then, run CalTrack for each of the datasets, every time pointing to the respective folder containing the Calcium_Traces.mat generated by the xlsx_to_mat() function.

Default downsampling for acquisition frequencies above 100 FPS

In order to enable CalTrack analysis to be conducted independently of the acquisition frequency used, if the acquisition frequency is larger than 100 FPS, the signal is automatically downsampled

to ~100 FPS and smoothed. This is only needed to get a good *diff* signal to obtain the start of the transients. Subsequently, the segmented beats contain the original signal (at the original acquisition frequency, to ensure no information is lost).

Segmentation based on stimulus times provided by the user

The function BeatSegmentation2.m performs beat segmentation based on the stimulus times provided by the user. The user must provide an excel file named “T0” than contains, for each cell, the frame number where the first electrical stimulus has been delivered to the cell.

This frame number is used to segment transients: the first transient is defined from T0 to T0+CL, and subsequently the second is defined at T0+CL to T0+2*CL, and so on. CL (cycle length) is computed as 1/pacing frequency.

The segmented transients are then averaged, and parameters are computed as described above.

By definition, this means that the reported t0 in the calcium parameters excel file is the distance (time) from the electrical stimulus (T0).

For Multi Cell the file T0 contains the stimulus times for each video (each cell in the same video is considered to have the same T0).

Multi Cell

The “Multi Cell” algorithm works in a similar way to the “Single Cell” algorithm.

In this scenario, videos that contain multiple cells must be analyzed; for each cell, the user aims to extract their multi-beat fluorescent trace. Subsequently, from each individual trace, single transients are identified, averaged, and quantified.

As for the “Single Cell” algorithm, the original movies may be in different formats including .tif image stacks. For the latter, the user needs to download Bio-Formats as reported above. All other formats, with the exception of .m4v files must be converted to .tif format via the algorithm’s prompt system.

Scripts

1. CalTrack.m

This script is run if the user opts to extract traces from videos in the input dialog box, as described above.

The script converts video files into .tif image stacks (via the script "convert_to_tif") and reads these into a MatLab structure that contains a multi-beat fluorescent trace for each cell in the image stack. Note that this script is set to read .m4v and .tif files without converting, however, all other video files require conversion via the script "convert_to_tif".

Given one image stack, cells are segmented by multi-step image processing.

The user can visualize the segmented cells superimposed onto the original image, and their corresponding multi-beat calcium curves (Figure 11). The user can then decide whether they want to discard any of the segmented cells for quality control, for example when cells haven't been adequately segmented, or when they do not display calcium transients. The multi-beat florescent trace of each cell is saved in the excel file "CalciumData_MultiCell", with cells in the same video file represented in columns and individual video files in separate sheets.

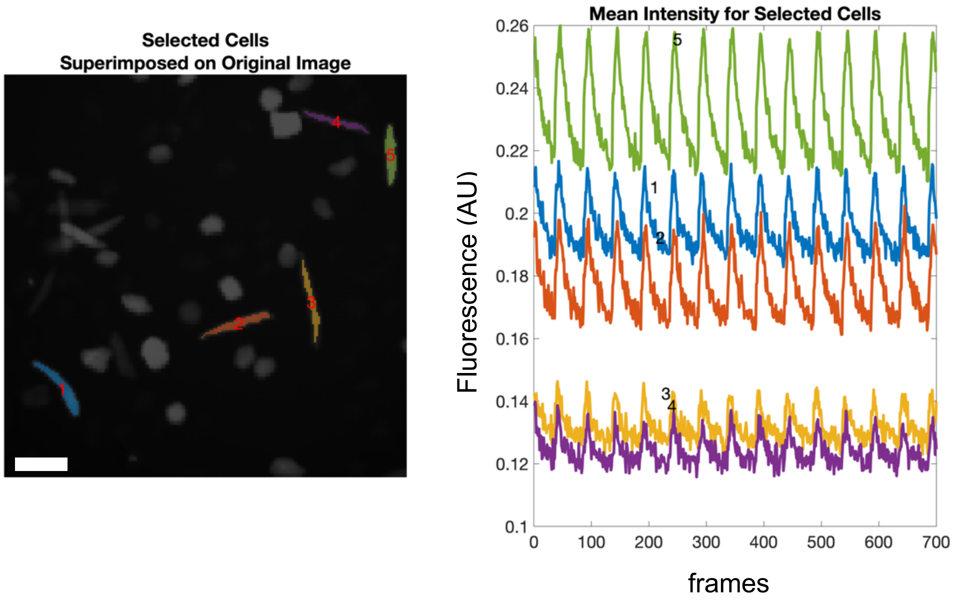

Figure 11. Segmented cells superimposed onto the original image (left) and their corresponding multi-beat calcium curves (right). The white bar is $100\ \mu m$.

2. DataAnalysis.m

The excel file from the script CalTrack.m is read by DataAnalysis.m.

For each cell in each video, single-beat transients are identified with the same algorithm described above for single cells. These are averaged for each cell and parameters are computed as for the single cell scenario. Individual transient analysis is not offered by the algorithm, since low magnification videos are more prone to noise. Averaging signals smoothes noise and therefore their analysis provides more accurate measurements.

The algorithm subsequently performs photobleach correction (if opted by the user) followed by beat segmentation and averaging. If photobleach correction is performed, corrected traces are plotted against their respective original ones and the user can decide to discard any cell if the correction is not adequate.

After the averaging process, segmented transients for each cell in each video are plotted (Figure 12) and the user can decide to discard any cell in any video for quality control purposes.

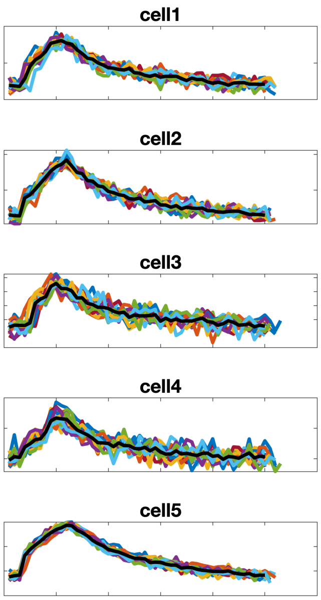

Figure 12. Example figure for one video. The user can visually check that the segmentation of the transients for each cell in the video was successful. Black traces represent average transients.

For each cell in each video, the average transient and parameters are saved in excel files (in the average transient file, each video file is represented in a sheet and individual cells in video files are represented in individual columns; for the measurements file, each cell's measurements are reported in one row, and are organized by video number (first column) and cell number (second column). For visualization purposes, the measurement file is also exported in a different format where each video's parameters are saved in a separate sheet. This is the file read by PlotParameters.m.

One important difference between the single cell and multi cell recordings is that, given the level of noise in the multi cell recordings at lower magnification, some additional signal processing may be needed.

In particular, signal y is substituted with its moving mean value (considering 5 points); the *diff* is computed using this new signal. The *diff* signal is also processed through the moving mean so that peaks can be identified more robustly (Figure 13). The moving mean is only used to facilitate the

identification of the single transients. This is then applied to the original signal, which is saved and analyzed.

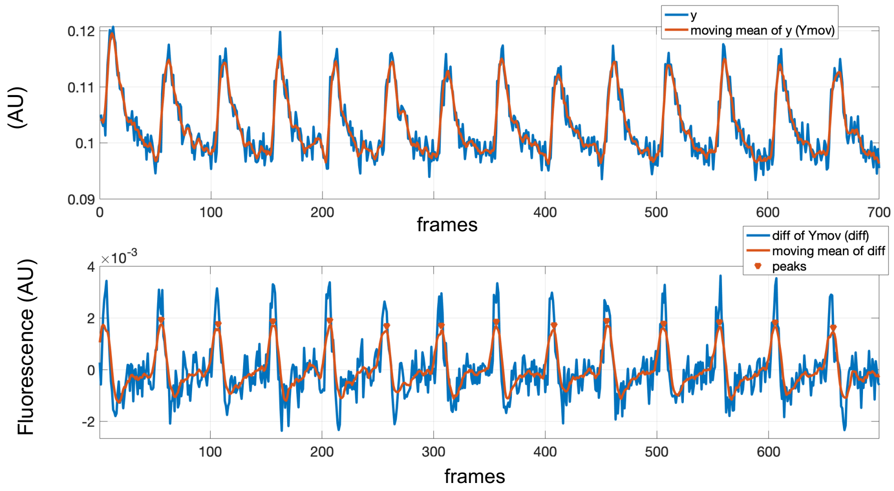

Figure 13. Comparison between y and diff signals with their corresponding moving means.

Similar to the "Single Cell" algorithm, only the subset of frames chosen by the user are analyzed. Four types of traces are not automatically analyzed but saved in separated excel files for further analysis:

- Transients with high noise where peaks cannot be robustly identified.
- Experiments where there is non-adherence to pacing or a specific beat rate.
- Traces for which non-specific error occurs (rare and can be caused by a number of technical inconsistencies with the initial acquired image stack).
- Traces for which error in computing the parameters occurs.

3. PlotParameters.m

This script is only run if the user opts to plot the results following analysis. The script reads the excel files exported from DataAnalysis.m, and plots for each cell in each video the average transient and corresponding parameters. This allows the user to visually check that each parameter (and fit) was computed correctly.

Functions

Below we outline the functions that support scripts CalTrack and DataAnalysis (descriptions provided in the "Single Cell" section above):

- convert_to_tif
- single_stack_loader
- BeatSegmentation
- PhotoBleachCorrection
- parameters.m
- intersections.m

Additional analysis features as described for Single Cell can be applied here

- Default downsampling for acquisition frequencies above 100 FPS
- Segmentation based on stimulus times provided by the user

Please note that functions FR_to_calcium.m and xlsx_to_mat.m only apply for Single Cell.
